# The strand-biased mitochondrial DNA methylome and its regulation by DNMT3A

**DOI:** 10.1101/gr.234021.117

**Published:** 2019-10

**Authors:** Xiaoyang Dou, Jerome D. Boyd-Kirkup, Joseph McDermott, Xiaoli Zhang, Fang Li, Bowen Rong, Rui Zhang, Bisi Miao, Peilin Chen, Hao Cheng, Jianhuang Xue, David Bennett, Jiemin Wong, Fei Lan, Jing-Dong J. Han

**Affiliations:** 1 Key Laboratory of Computational Biology, CAS Center for Excellence in Molecular Cell Science, Collaborative Innovation Center for Genetics and Developmental Biology, Chinese Academy of Sciences–Max Planck Partner Institute for Computational Biology, Shanghai Institutes for Biological Sciences, Chinese Academy of Sciences, Shanghai 200031, China;; 2 University of Chinese Academy of Sciences, Beijing 100049, China;; 3 Peking-Tsinghua Center for Life Sciences, Academy for Advanced Interdisciplinary Studies, Center for Quantitative Biology (CQB), Peking University, Beijing 100871, China;; 4 Liver Cancer Institute, Zhongshan Hospital, Fudan University, Key Laboratory of Carcinogenesis and Cancer Invasion, Ministry of Education, Key Laboratory of Epigenetics, Shanghai Ministry of Education, and Institutes of Biomedical Sciences, Fudan University, Shanghai 200032, China;; 5 Shanghai Key Laboratory of Regulatory Biology, Institute of Biomedical Sciences and School of Life Sciences, East China Normal University, Shanghai 200241, China;; 6 The State Key Laboratory of Molecular Biology, Institute of Biochemistry and Cell Biology, Chinese Academy of Sciences, Shanghai 200031, China;; 7 Rush Alzheimer's Disease Center, Rush University Medical Center, Chicago, Illinois 60612, USA

## Abstract

How individual genes are regulated from a mitochondrial polycistronic transcript to have variable expression remains an enigma. Here, through bisulfite sequencing and strand-specific mapping, we show mitochondrial genomes in humans and other animals are strongly biased to light (L)-strand non-CpG methylation with conserved peak loci preferentially located at gene–gene boundaries, which was also independently validated by MeDIP and FspEI digestion. Such mtDNA methylation patterns are conserved across different species and developmental stages but display dynamic local or global changes during development and aging. Knockout of *DNMT3A* alone perturbed mtDNA regional methylation patterns, but not global levels, and altered mitochondrial gene expression, copy number, and oxygen respiration. Overexpression of *DNMT3A* strongly increased mtDNA methylation and strand bias. Overall, methylation at gene bodies and boundaries was negatively associated with mitochondrial transcript abundance and also polycistronic transcript processing. Furthermore, HPLC-MS confirmed the methylation signals on mitochondria DNA. Together, these data provide high-resolution mtDNA methylation maps that revealed a strand-specific non-CpG methylation, its dynamic regulation, and its impact on the polycistronic mitochondrial transcript processing.

Intense investigation of epigenetic factors continues to shape our understanding of genomic regulatory dynamics, and mitochondria activity has been implicated as a key factor in this via direct and indirect control of numerous epigenetic enzymes (Matilainen et al. 2017). However, the possibilities and implications of mitochondria-autonomous epigenetic regulation have been largely overlooked and controversial (D'Amico et al. 2017), but are of great potential importance because alterations to mtDNA expression or coding sequences are directly causative of premature aging (Trifunovic et al. 2004) and metabolic and neurodegenerative diseases (Schapira 2012).

Discovery of mtDNA methylation was reported in the 1970s using radiolabeling (Nass 1973). Subsequently, a series of studies showed the existence of relatively low-level methylation of mtDNA compared with nuclear DNA (Shmookler Reis and Goldstein 1983; Pollack et al. 1984; Dzitoyeva et al. 2012). Later, mtDNA methylation was invariably detected by antibody-based techniques such as MeDIP (Ghosh et al. 2014; Devall et al. 2017), both CpG and non-CpG methylation were observed on mtDNA, and they are in a strand-biased pattern (Bellizzi et al. 2013). Conversely, by focusing only on the CpG or the average methylation level of all Cs of mtDNA, some studies showed the absence of mtDNA methylation (Supplemental Fig. S1A,B; Supplemental Table S1; Dawid 1974; Maekawa et al. 2004; Liu et al. 2016; Mechta et al. 2017; Owa et al. 2018). Strong indirect support for the methylation of mtDNA has been provided by the presence of DNA methyltransferases in mitochondria. Mitochondrial fractions were also found to contain DNA methyltransferase activity (Nass 1973). A long isoform of the DNMT1 has a mitochondrial targeting sequence (MTS) and is found to enter mitochondria (Shock et al. 2011), and although canonical MTSs are not present in the primary sequences of de novo methyltransferases DNMT3A and DNMT3B, evidence that they are present in mouse mitochondria was reported (Chestnut et al. 2011; Bellizzi et al. 2013; Wong et al. 2013). TET1 and TET2 were reported to localize in the mitochondria of 3T3 cells (Chen et al. 2012), and the translocation of DNMTs and TETs to mitochondria is tissue-specific (Bellizzi et al. 2013). The shortest isoform of DNMT1 was recently reported to localize in mitochondria of H1229 cells, and perturbation of *DNMT1* affected mtDNA methylation (Saini et al. 2017). Recently, a genome-scale profile of methylation and hydroxymethylation of mtDNA from analysis of public MeDIP-seq has been reported (Ghosh et al. 2014, 2016). However, MeDIP-seq uses anti-methylcytidine antibody to enrich for methylated DNA fragments, so it cannot reach base resolution like whole-genome bisulfite sequencing (WGBS). In addition, evidence of specific mtDNA-target methylation sites by these enzymes has not been shown, and with the absence of a precise map of mtDNA methylation, the existence of mtDNA methylation has remained ambiguous and debated (Supplemental Fig. S1A,B; Supplemental Table S1; Hong et al. 2013; van der Wijst and Rots 2015; Mechta et al. 2017; Owa et al. 2018).

## Results

### Strand-specific mapping of mtDNA methylation

**Figure 1.**
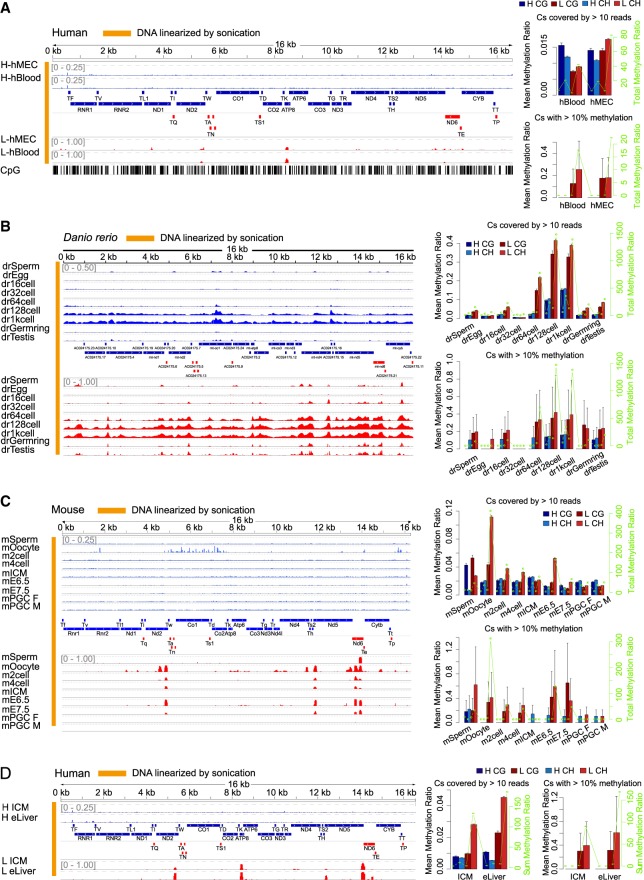
Strand-specific mapping of mtDNA methylation. (*A*, *left*) BS-seq (WGBS) methylation profiles of linearized mtDNA in human B cells (GSM922328 under GSE37578) and mammary epithelial cells (MEC; GSM721195 under GSE29127). The annotations between the H and L strand methylation maps show positions of mtDNA elements: In blue are polypeptide genes, rRNAs and tRNAs coded on the H strand and using L strand as template; in red are polypeptide genes, rRNAs and tRNAs coded on the L strand and using H strand as template. (*Right*) Mean (*left y*-axis) and total (*right y*-axis) mtDNA methylation of CG and CHs on H and L strand, respectively. The mean methylation level is the average methylation level of C sites covered by >10 reads. The total methylation level is the sum of methylation level of C sites covered by >10 reads. We only considered C sites covered by at least 10 reads, with all such C sites (*top*) or part of them with >10% methylation (*bottom*) summarized and the stringent cutoff (10%) is used to avoid arguments that some lowly methylated Cs are attributable to background noise. Error bars represent SEM. (*B*, *left*) BS-seq (WGBS) methylation profiles of linearized mtDNA in zebrafish sperm, oocyte (eggs), cleavage-stage embryos at the 16-cell, 32-cell, and 64-cell stages, early-blastula 128-cell stage, midblastula stage (MBT) 1000-cell stage (1kcell), the gastrula stage at the germ ring (Germring), and testis from an inbred TU strain (Testis) during early embryogenesis (GSE44075) on the H and L strand. Blue tracks show methylation levels of the H strand (scale from 0 to 0.5), and red tracks show L-strand methylation (scale from 0 to 1.0). Annotations are as in *A*. (*C*, *left*) BS-seq (WGBS) methylation profiles of linearized mtDNA in mouse sperm, oocyte, and early stage embryos including two-cell and four-cell cleavage stages, early ICM, E6.5 embryos, E7.5 embryos, and the primordial germ cells (PGCs) from E13.5 male and female embryos (GSE56697) on the H and L strands, respectively. Blue tracks show methylation levels of the H strand (scale from 0 to 0.25), and red tracks show L-strand methylation (scale from 0 to 1.00). Annotations are as in *A*. (*D*, *left*) BS-seq (WGBS) methylation profiles of linearized mtDNA in mouse late blastocyst (inner cell mass [ICM]) and human post-implantation embryonic liver (eLiver) (GSE49828) on the H and L strands. Blue tracks show methylation levels of the H strand (scale from 0 to 0.25), and red tracks show L-strand methylation (scale from 0 to 1.00). Annotations are as in *A*.

Focusing only on CpGs by using primers designed to capture CpGs and assuming CpH are unmethylated, Hong et al. (2013) found very low methylation level on CpGs. We reanalyzed the BS-seq data sets used in their paper and found that indeed the CpG methylation is low (Supplemental Fig. S1C). However, when summarization is carried out in a strand-specific manner, we observed high non-CpG methylation specifically in L strand (Fig. 1A). Consistently, when we focused on the highly methylated regions (>10% methylation, i.e., setting <10% methylation to 0 as background), the highly methylated C sites are exclusively located at the non-CpG sites in L strand (Fig. 1A; Hong et al. 2013; Liu et al. 2016; Mechta et al. 2017; Matsuda et al. 2018; Owa et al. 2018). We then used strand-specific mapping to examine more published data sets on early development in zebrafish (Fig. 1B), mouse (Fig. 1C), and human (Fig. 1D) to see if the high L-strand-biased mtDNA non-CpG methylation is developmentally regulated. Indeed, in all three species, it is dynamically and developmentally regulated. Although high L-strand non-CpG methylation peaks are largely conserved and localized to gene boundaries, oocytes showed strong and pervasive methylation across the L strand, but mouse inner cell mass (ICM) and primordial germ cells (PGCs) displayed no methylation (Fig. 1C). Meanwhile, local but not global differences exist between embryonic day 6.5 and 7.5 mice (Fig. 1C), and between human ICM and embryonic liver (Fig. 1D). We found that removing sequences with Nuclear Mitochondrial Sequences (NUMTs) from public data sets did not substantially alter methylation quantification (Supplemental Fig. S1D,E; see below).

### Confirmation of mtDNA methylation during human brain aging and in human cell lines

Human prefrontal cortex (PFC) was initially chosen exploratively because PFC is an important tissue that contains much higher mtDNA copies and relatively abundant CpH methylation. In addition, brain mitochondria can contribute to the pathogenesis of various neurodegenerative diseases such as Alzheimer's, Huntington's, and Parkinson's disease, making the study of mitochondrial DNA methylation in human brain highly relevant.

**Figure 2.**
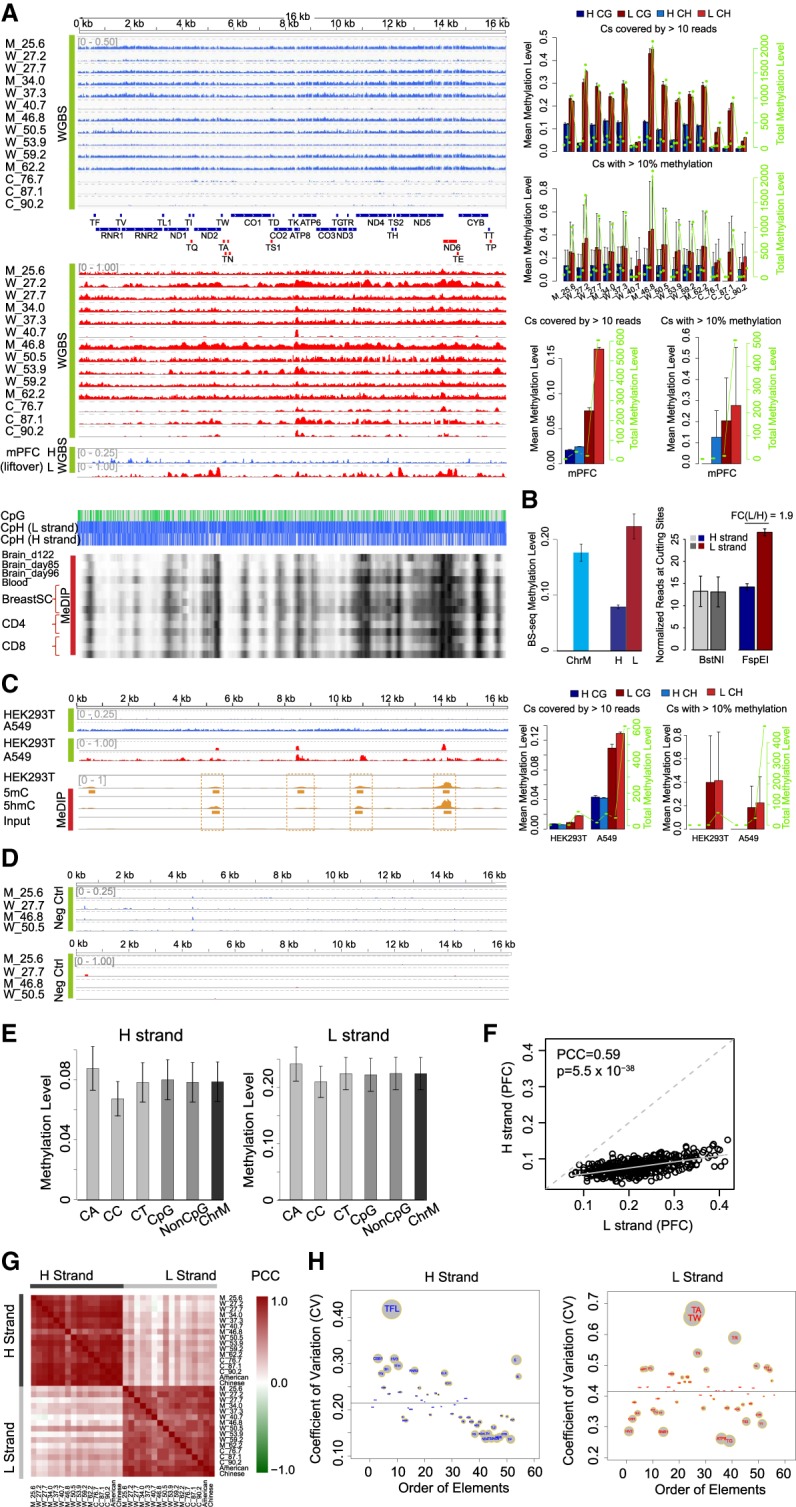
Human mtDNA methylation is highly conserved between individuals, populations, and cell types in a strand-biased fashion. (*A*, *left*) BS-seq methylation profiles of mtDNA in human PFC and BS-seq (WGBS) methylation profiles of mtDNA in mouse (GSE33722) PFC samples on the H and L strands, and MeDIP-seq methylation profiles of mtDNA in human brain, blood, breast stem cell, CD4, and CD8 (GSE16368). Different tracks labeled M, W, and C are individual PFC samples (Supplemental Table S3) ranked by their mean ages. Blue tracks show methylation levels of the H strand (scale from 0 to 0.5 for hPFC, and 0 to 0.25 for mPFC), and red tracks show L-strand methylation (scale from 0 to 1). Annotations are as in Figure 1. (*B*) Mean methylation levels of all cytosines on ChrM, H, and L strand, respectively, in PFC samples. Error bars represent SEM among 14 PFC samples (*left*). The normalized reads at BstNI and FspEI digestion sites on mtDNA H and L strands in mouse PFC samples (GSE33722), respectively (*right*). (*C*, *left*) BS-seq methylation (WGBS) profiles of mtDNA in HEK293T and A549 on the H and L strands with WGBS, and MeDIP-seq methylation profiles of mtDNA in HEK293T with 5mC and 5hmC MeDIP-seq and the corresponding input. The *right* panel shows summary statistics as annotated in Figure 1. (*D*) BS-seq methylation profiles of PCR-amplified mtDNA from four human PFC samples as negative controls. (*E*) Mean methylation of different cytosine dinucleotides on the H and L strands in 14 human PFC samples. Error bars represent SEM. (*F*) Scatter plot of mean methylation on CpG sites between the L and H strand in 14 human PFC samples, and each dot represents one CpG site. (*G*) Heatmap of correlation (PCC) between each pair of human brain PFC samples on the H and L strands. (*H*) Coefficient of variation (CV) of methylation on each functional element across all samples of PFC on the H or L strand. The size of each element's circle is scaled according to the absolute difference between its CV and the mean of all CVs, which is indicated by the horizontal dashed lines.

Using a mitochondria enrichment method (Methods; Supplemental Fig. S2A,B; Supplemental Table S2), mtDNA was isolated from human PFC from 98 normal donors that cover 23–92 yr of age (Supplemental Table S3) and cell lines. We further used WGBS to generate single-base mtDNA methylation data in human PFC (Fig. 2A,B) and human and mouse cell lines, and performed parallel MeDIP in human cells (Fig. 2C). Unmethylated lambda DNA was used as a spike-in control, and two PCR products of mtDNA were used as an unmethylated mtDNA negative control to ensure bisulfite conversion efficiency (Fig. 2D). Sequences were strand-specifically mapped (Supplemental Information) and analyzed to ensure all cytosines were mapped to 10× saturation (Supplemental Fig. S2C,D). Several experimental approaches to deplete nuclear DNA were used to validate that mapping mtDNA from samples containing nuclear DNA did not lead to significant read mapping from nuclear sequences (Methods).

Consistent with the methylation profiles observed (Fig. 1A–D), and in particular similar to a previously published mouse brain WGBS data displaying extensive L-strand methylation (Fig. 2A, “mPFC” track), we observed a twofold to fivefold higher methylation level on the L strand in human PFC (Fig. 2B) independent of its coverage difference (Supplemental Fig. S2E,F), coverage cutoff (Supplemental Fig. S2G), and mapping method used (Supplemental Fig. S2H,I). In all PFC samples, we observed an extensive L-strand-biased mtDNA methylation, particularly high in non-CpGs; the H strand had an average methylation of only 8%, whereas the L strand was 23% methylated (Fig. 2A,B; Supplemental Table S4). The strong methylation peaks are also detected in published human brain bisulfite-independent MeDIP-seq data, although no strand specificity can be analyzed by the MeDIP-seq technique (Fig. 2A, MeDIP heatmaps). No methylation was observed in negative controls of PCR-amplified mtDNA from a subset of the same samples (Fig. 2D) and in the spike-in lambda DNA (Supplemental Fig. S2J), assuring complete bisulfite conversion occurred.

To confirm this strand-biased methylation, we generated BS-seq maps for three commonly used cell lines and observed similar L strand–associated high methylation: HEK293T (strand average 0.6% and 1.8%, peak average 0.72% and 30%, on H and L strands), A549 (4.2% and 12.8%, peak average 4.3% and 39% on H and L strands), and mESC (1.1% and 1.4%, peak average 1.6% and 22% on H and L strands) (Fig. 2C; Supplemental Fig. S6L; Supplemental Table S4). The MeDIP and BS-seq peaks we observed are similarly observed in approximately 40 other MeDIP-seq data sets (examples are shown in Fig. 2A) (Ghosh et al. 2014). For HEK293T cells, we also used bisulfite-independent 5mC and 5hmC MeDIP-seq to map methylation. Although MeDIP-seq tags cannot be mapped strand specifically, we observed a MeDIP-seq profile highly correlated with the BS-seq L-strand profile (5mC MeDIP vs. BS-seq, PCC = 0.34; *P* = 2.5 × 10^−10^). In particular, 5hmC MeDIP-seq profile showed a much higher correlation with BS-seq (PCC = 0.46, *P* = 4.9 × 10^−19^) than 5mC MeDIP with highly consistent methylation peak loci, suggesting mtDNA methylation—in particular with L-strand methylation peaks—detection is not dependent on BS-seq per se, or any particular method (Fig. 2C).

### Strand-biased CpH rich mtDNA methylation

We observed significant non-CpG methylation on both strands at a similar level to that of CpG methylation in PFC samples (Fig. 2E). Even at symmetrical CpGs, the methylation is biased toward the L strand, where the mirroring Cs on the H strand are still lowly methylated compared with L, thus ruling out local secondary structure of a double-stranded DNA as causing detection of high L-strand methylation (Fig. 2F) in our studied samples. However, we cannot rule out the effects of secondary structure of mtDNA in affecting methylation level detection when using bisulfite treatment in certain contexts, as reported by Liu et al. (2016).

Globally, there is a high correlation across samples in both H and L strands (mean pairwise Pearson correlation coefficient [PCC] = 0.78 and 0.62 for H- and L-strand methylation in all human samples, respectively; FDR = 0 based on 1000 read mapping permutations) (Fig. 2G); however, methylation landscapes are poorly correlated between strands (Fig. 2G), underscoring the strand-specific nature of mtDNA methylation.

We examined cross-sample similarities in PFC methylation profiles at mitochondria genomic elements and found that adjacent regions had more similar patterns overall, supporting the nonrandomness of the patterns (Supplemental Fig. S3A). Unsupervised hierarchical clustering showed that the genomic elements of the same type (coding genes, tRNAs, rRNAs, or regulatory elements) have highly correlated methylation (Supplemental Fig. S3B).

The L strand, in addition to showing higher methylation, showed the most variable methylation patterns, predominantly in coding regions. *MT-ATP8* contained the largest (Fig. 2A) and most conserved methylation peak on the L strand across samples (Fig. 2H). Another highly methylated peak on the L strand was found at the border between the *MT-ND5* (H strand–encoded) and *MT-ND6* (L strand–encoded) (Fig. 2A). Protein-coding genes were the most highly methylated gene class, especially for the L strand (Supplemental Fig. S3C). On both strands there was a general increase in methylation levels in a clockwise manner from the D-Loop (Supplemental Fig. S3D), coinciding with a clockwise decrease in corresponding transcript abundance (Supplemental Fig. S3E). Regulatory elements also showed diversity in methylation between samples and elements (Fig. 2H); for example, *MTTFL* showed high variance across samples (CV of about 0.4–0.5) on the H strand. However, on the L strand, tRNAs showed similar, if not higher, variance (CV up to 0.60 at *TA* and *TW*). Protein-coding genes showed much lower variance on both strands and were the least variable class on the H strand (Fig. 2H).

### Controlling for confounding factors of mtDNA BS-seq methylation analysis

With >10× coverage on all cytosines on both strands (Supplemental Fig. S2C,D), we observed a roughly 18- to 114-fold higher read mapping coverage to the lowly methylated H strand in PFC. We also noted similar read mapping and methylation biases in HEK293T, A549 cells, and mouse oocyte (Supplemental Table S4; Zhao et al. 2014). In the same samples, no bias was present for nuclear DNA (Supplemental Fig. S4A–C). We further rule out the possibility of high L-strand methylation caused by special mtDNA secondary structure as has been assumed (Liu et al. 2016; Mechta et al. 2017; Owa et al. 2018) or as a consequence of read coverage bias (Supplemental Information).

A possible confounding factor in mtDNA methylation analysis comes from nuclear sequences closely resembling mtDNA (i.e., NUMTs), although these are typically hundreds to thousands of times less abundant than mtDNA and therefore unlikely to yield a significant proportion of reads. To address the possibility of contamination by NUMTs, we compared several mapping strategies and found that removing NUMT-matched reads contributed little to methylation level or profile (summarized in Supplemental Table S5 and exemplified by Supplemental Fig. S4D). We calculated the methylation level on NUMTs overlapping and non-NUMTs overlapping C sites and observed they had almost the same strand bias and level of methylation (summarized in Supplemental Table S6). Together, this shows there is no need to remove NUMT-matching reads and is consistent with other studies that find that the great majority of NUMTs come from mitochondrial DNA (Li et al. 2012; Hong et al. 2013). Therefore, in the following analysis of functional associations, we used all reads that can be mapped to mtDNA.

### mtDNA methylation correlates with SNP density and decreases during aging

**Figure 3.**
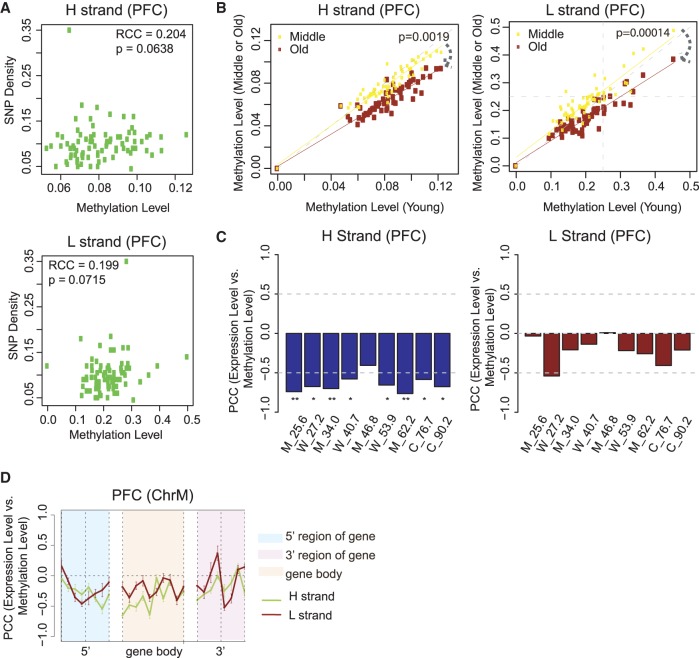
mtDNA methylation is correlated with SNP density, decreases during aging, and is negatively correlated with gene expression. (*A*) Spearman's rank correlation coefficient (RCC) between mean methylation 200 bp per bin and SNP density on the H and L strands. An SNP data set (SNP142) was downloaded from NCBI. ChrM is binned per 200 bp, and SNP density per bin is defined as the number of SNPs located in that bin divided by length of the bin (200 bp). Subsequently, correlation between SNP density and mean methylation on hPFC samples, per bin, was calculated. (*B*) Scatter plot of methylation on each of the functional elements annotated in MITOMAP between young and middle (or young and old) age groups on the H and L strands. Human brain samples are separated based on age: young (20–40 yr), middle (40–60 yr), and old (>60 yr). *P*-value between two lines is calculated based on a one-sided *t*-test. (*C*) Correlation between transcript abundance and mean methylation level within each sample on the H and L strand: (*) *P* < 0.05; (**) *P* < 0.01. (*D*) Correlation between transcript abundance and methylation within the sample (across genes) in the 5′ region, gene body, and 3′ region of H-coded genes in human PFC in 50-bp windows. The gene 5′ and 3′ regions are defined as the ±200 bp at the 5′ and 3′ end of genes except *MT-ATP6*, which overlapped with both *MT-ATP8* and *MT-CO3*, respectively. Error bars represent SEM across H-coded genes and nine PFC samples. Functional elements are shown in the legend.

We examined whether methylation level across mtDNA was associated with mutation. The incidence of mtDNA SNPs from dbSNP was positively, albeit not significantly, correlated with the methylation level (Fig. 3A), suggesting that mtDNA methylation similarly predisposes cytosine to mutation, as found in the nucleus. Consistent with previous reports, in PFC samples we found mitochondrial gene expression and copy number decreased and increased with age, respectively (Supplemental Fig. S5A,B). For both strands, age is associated with a general decrease in global methylation (Supplemental Fig. S5C). Further, methylation differences between old and young ages were highly significant, with methylation at most elements decreased in old age (*t*-test *P* = 1.9 × 10^−3^ for H strand and 1.4 × 10^−4^ for L strand) (Fig. 3B).

### mtDNA methylation negatively correlates with mitochondrial gene expression

Although mtDNA is transcribed in a polycistronic fashion, mRNA levels of individual genes differ substantially, reflecting differences in processing, stability, or turnover of each mRNA (Supplemental Fig. S3E). For RNA-seq analysis, similar to bisulfite sequencing, we ruled out the possibility of contamination by nuclear-derived transcripts in mRNA expression data (Supplemental Methods). We compared the associations of methylation to transcript abundance within each sample and observed a negative correlation between gene body methylation on both strands with transcript abundance (Fig. 3C).

On the L strand, methylation at the 5′ boundary of genes showed a negative correlation with transcript abundance, suggesting this position might be important to negative regulation of the corresponding transcript (Fig. 3D). On both strands, methylation of the gene body was generally negatively correlated with abundance, but this association was lost at gene 3′ boundaries and turned to positive correlation at L-strand gene 3′ boundaries (Fig. 3D). This suggests that DNA methylation on the L strand at gene boundaries is associated with decreased processing of the gene after it, but with increased processing of the gene in front of it, which suggests that an increased pausing of the processing machinery might occur at the methylation site.

DNA methylation in the nucleus undergoes hyper- and hypomethylation in waves coinciding with key early developmental changes. Using recent WGBS data, we found that global mtDNA methylation followed a partially similar trend with nuclear DNA in early embryogenesis, with a PCC of 0.51 (*P* = 0.17) in mouse germ cells up to embryonic and primordial germ cell stages (Fig. 1C; Supplemental Fig. S5D,E). Mouse mtDNA methylation followed the pattern of high oocyte methylation followed by hypomethylation after fertilization and diverged with nuclear DNA methylation at embryonic day 6.5 (Supplemental Fig. S5E). This indicates mtDNA methylation is dynamic and may partially share regulatory mechanisms with the nucleus. However, mitochondria lack similarity in the context of chromatin, and coordination during development between DNMTs and other factors are not expected to be identical (Supplemental Information).

### mtDNA methylation, especially between *MT-ND2* and *MT-CO1*, is modulated by DNMT3A

**Figure 4.**
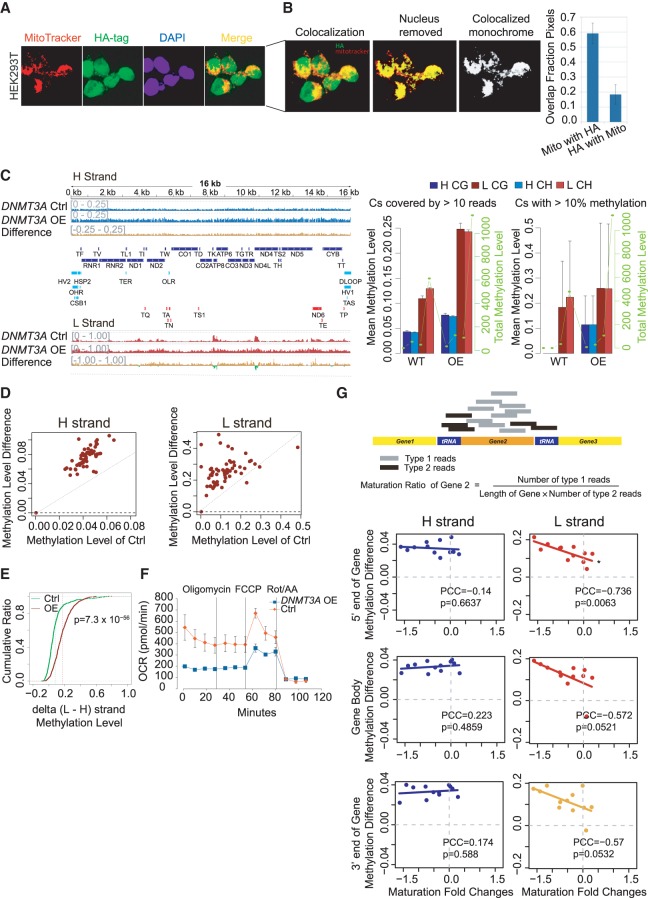
Presence of DNMT3A in mitochondria and effects of *DNMT3A* overexpression on mtDNA methylation and transcript processing. (*A*) Immunofluorescence staining of HA-tagged DNMT3A. Cells were exposed to MitoTracker Orange (red) to label mitochondria and anti-HA with *DNMT3A*-transfected cells (green) and DAPI (blue) to label nuclei. Yellow pixels in the *Z-*stack composite indicate sections in which DNMT3A colocalized with mitochondria, showing that DNMT3A localized to both nucleus and mitochondria. (*B*) Overlap of mitochondria fluorescence with DNMT3A-HA-tagged fluorescence among pixels passing a stringent background threshold to remove correlation contributed by background signal. (*C*, *left*) mtDNA methylation profiles after *DNMT3A* overexpression (*DNMT3A* OE) on the H and L strands. Blue tracks show methylation levels of the H strand (scale from 0 to 0.25) or L strand (scale from 0 to 1), and red (positive) or green (negative) tracks show methylation difference. Annotations and summary statistics are as in Figure 1. (*Right*) Mean (*left y*-axis) and total (*right y*-axis) mtDNA methylation of CG and CHs on the H and L strands, respectively. (*D*) Scatter plot between the mean methylation level of 63 functional elements of control samples (*x*-axis) and corresponding *DNMT3A* OE samples (*y*-axis, *DNMT3A* OE) on the H and L strands. (*E*) Cumulative curve of methylation level difference between L and H strands after sampling 1000 paired Cs under *DNMT3A* OE. The *P*-value for the difference between *DNMT3A* OE and control was calculated with a one-sided *t*-test. (*F*) Oxygen consumption of *DNMT3A* OE with control A549 cells. (*G*, *upper*) Graphic illustration of maturation ratio calculation. (*Lower*) Scatter plot of maturation fold changes (FC) against H- and L-strand methylation difference of 5′ end (±200 bp of TSS), gene body, and 3′ end (±200 bp of TTS) of gene comparing *DNMT3A* OE with control. (*) Negative PCC with *P* < 0.01.

DNMT3A has been reported to localize to mitochondria and is required for maintenance of CpH methylation in neurons in vivo (Guo et al. 2014), and so to investigate whether mtDNA methylation is actively deposited by known methyl writers, we first confirmed that DNMT3A can be detected with antibodies recognizing HA-tagged DNMT3A or endogenous DNMT3A (Fig. 4A; Supplemental Fig. S6A). Using purified mitochondria fractions, western blot showed endogenous DNMT3A localized to the mitochondria and increased and decreased proportionally to overall DNMT3A levels when overexpressed or knocked down (Supplemental Fig. S6B), which indicates specificity of detection. Specific detection was also supported by western blots of mitochondria fractions in *DNMT3A* knockout (KO) and wild-type (WT) HEK293T cells and mESCs (Supplemental Fig. S6C,D). To detect localization using 3D confocal microscopy, *DNMT3A* was modified to express a C-terminal HA tag, and anti-HA fluorescence was abundant in both nucleus and mitochondria, as marked by MitoTracker dye (Fig. 4A). A conservative estimate of ∼60% of MitoTracker overlapped with DNMT3A-HA, whereas ∼20% of DNMT3A-HA localized in mitochondria rather than nuclei (Fig. 4B). We then profiled the methylome by WGBS in *DNMT3A* KO and WT HEK293T cells (Supplemental Fig. S6F). A major peak between *MT-ND2* and *MT-CO1* was abolished by *DNMT3A* KO (Supplemental Fig. S6F) and there was a ∼20% increase in mtDNA copy number (Supplemental Fig. S6G). Over all methylation peaks, the methylation level determined by BS-seq was decreased by 10% in *DNMT3A* KO compared with WT (Supplemental Fig. S6H).

To exclude bias introduced by bisulfite treatment, we used a methylation-sensitive restriction enzyme, FspEI, to validate the decrease of mtDNA methylation in *DNMT3A* KO. Consistently, we observed a significant decrease in the number of digested mtDNA fragments from *DMNT3A* KO, compared with control HEK293T cells, upon FspEI digestion (Supplemental Fig. S6H). Concordantly, we also observed a global increase of mitochondrial gene expression, normalized by mtDNA copy number (Supplemental Fig. S6I,J). The mitochondrial oxygen consumption also increased in the *DNMT3A* KO cells (Supplemental Fig. S6K). These data suggest that a significant loss of the methylation between *MT-ND2* and *MT-CO1* leads to changes in mitochondrial gene expression possibly owing to the polycistronic nature of transcription from the circular mtDNA genome and consequently mitochondrial function.

Because DNMTs do not function independently, having partial redundancy and functional compensation between each other (Supplemental Fig. S6E), we hypothesized that this is responsible for the lack of global mtDNA methylation change in *DNMT3A* KOs. Because most cells cannot proliferate after loss of *DNMT1*, we analyzed WGBS data in mESCs, which can tolerate *Dnmt1* loss, and compared mtDNA methylation between WT, *Dnmt1*-KO, *Dnmt3a*/*Dnmt3b* KO double KO, and *Dnmt1*/*Dnmt3a*/*Dnmt3b* triple (TKO) (Supplemental Fig. S6L). We found that only in TKOs was mtDNA methylation globally reduced (Supplemental Fig. S6L). Mitochondrial-encoded gene expression in the TKO mESCs was also globally increased, as expected (Supplemental Fig. S6M).

To avoid confounding compensatory effects of other DNMTs in *DNMT3A* loss, we examined the overexpression of *DNMT3A*. WGBS and RNA-seq was performed in the *DNMT3A* OE A549 and parental cells, and we observed *DNMT3A* OE increased global mtDNA methylation level (Fig. 4C,D) and strand bias (Fig. 4E). Thus knockout and overexpression of *DNMT3A* both reduced the strand difference in methylation levels, further confirming the strand-specific nature of mtDNA methylation (Supplemental Fig. S6N,O) and decreased global transcript abundance (Supplemental Fig. S7A), but slightly increased (∼6%) mtDNA copy number (Supplemental Fig. S7B). This and the decreased mtDNA methylation upon *DNMT* KOs strongly indicate that detected methylation was not a result of technical artifacts of bisulfite sequencing mtDNA, because such artifacts would be present in controls, OE, and TKO samples equally. As expected with the global down-regulation of mitochondrial gene expression by *DNMT3A* OE (Supplemental Fig. S7A), mitochondria oxygen consumption rate decreased in *DNMT3A* OE cells (Fig. 4F), consistent with the opposite effect in *DNMT3A* KO (Supplemental Fig. S6K). Together this not only confirmed the association of mtDNA methylation with transcript abundance (Supplemental Fig. S7C), but also showed causality of mtDNA methylation perturbation to transcript abundance changes. Because we did not observe any significant expression changes for nuclear-encoded mtDNA transcription factors under either *DNMT3A* KO or OE (Supplemental Fig. S7D,E), the mtDNA transcription regulation by *DNMT3A* is unlikely to be indirectly mediated by these nuclear-encoded factors (Supplemental Information).

We investigated the effect of methylation change at specific loci on nearby gene expression. Given that there is nonuniform mitochondrial gene expression (Supplemental Fig. S3E) and expression changes of different genes from the same transcripts upon aging (Supplemental Fig. S5F), or *DNMT3A* OE (Supplemental Fig. S7F), and the specific links found between gene boundary, and gene body methylation, and transcript abundance (Fig. 3D), we hypothesized that changes in mtDNA methylation at individual gene vicinities would affect processing of polycistronic transcripts into mature mRNAs. To test this, we performed Ribominus RNA-seq of the *DNMT3A* OE cell lines. To quantify differences between reads corresponding to processed transcripts and unprocessed (i.e., reads crossing a gene boundary), we defined a “maturation ratio” as a measure of transcriptional processing at each gene. Maturation ratio is calculated as the number of fragments mapped within a gene body divided by gene length, then divided by the number of fragments mapped across the gene boundary site (5′ plus 3′ end of a gene) (Fig. 4G, upper). We found a global decrease of maturation ratios in *DNMT3A* OE (Fig. 4G; Supplemental Fig. S7G). Unlike with the changes in transcript abundance (Supplemental Fig. S7F), which showed no correlation or slight positive correlation with 5′ gene boundary methylation changes, maturation changes were strongly negatively correlated with 5′ gene boundary methylation changes on the L strand, and to a lesser extent negatively correlated to methylation changes across the gene body and the 3′ of the gene across different genes (Fig. 4G). Together, this suggests an increase in L-strand methylation at gene boundaries, especially at the 5′ end, is particularly associated with a decrease of the individual gene's maturation or processing from a large polycistronic transcript derived using the L-strand template.

### Mitochondrial DNA methylation confirmation by HPLC-MS

**Figure 5.**
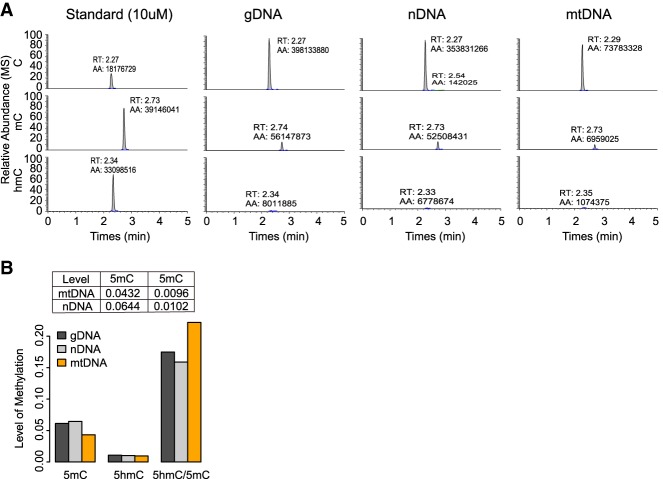
Mouse brain mtDNA methylation level detected by HPLC-MS. (*A*) C, 5mC, and 5hmC peaks detected by HPLC-MS on total genomic DNA (gDNA), nuclear DNA (nDNA), and purified mtDNA, analyzed in parallel with C, 5mC, and 5hmC standards. (RT) HPLC retention time in minutes; (AA) area of peak. (*B*) 5hmC and 5mC level relative to all Cs on total gDNA, nDNA, and purified mtDNA in WT mouse brain (Methods).

To further confirm the existence of mtDNA methylation was not attributable to unique technical artifacts found with bisulfite treatments, we performed HPLC-MS on genomic DNA (gDNA), nuclear DNA (nDNA), and mtDNA extracted from the same mouse brain samples in parallel to C, 5hmC, and 5mC standards. HPLC-MS showed the mouse brain mtDNA contained a high level of 5mC, but low levels of 5hmC (Fig. 5A,B). In addition, we estimated the lower bound of 5hmC on mtDNA as 0.84%, and the maximum of 5hmC and 5mC in the mouse brain are detected (0.96% and 4.32%) assuming there is no contamination of nDNA in mtDNA component (Methods).

## Discussion

Here, we have shown that mtDNA methylation can be reliably detected. This was supported by low false detection in negative controls, highly similar results between independent MeDIP-seq and BS-seq experiments, and complimentary results in reanalyses of published data sets generated using different bisulfite sequencing preparation methods. Using a strand-specific analysis pipeline, mtDNA methylation detection is robust across a wide range of cell and tissue types, shows clear peak patterns, and substantial strand-biased and non-CpG methylation. Perturbation of *DNMT3A* clearly affected mtDNA methylation, arguing against the possibility of methylation detection being random or attributable to technical artifacts.

Some reports indicated low or absent mtDNA methylation in BS-seq data and proposed bisulfite conversion is affected by the secondary structure of circular mtDNA because of low detected mtDNA methylation if it was linearized via restriction digestion (Hong et al. 2013; Liu et al. 2016; Mechta et al. 2017). However, these reports focused on only lowly methylated CpGs and did not perform strand-specific analysis. It is possible that focusing only on CpGs, neglecting non-CpGs, and combining reads of both strands for mapping methylation—especially because sequence coverage on the lowly methylated H strand is much higher than L strand—misled to conclusions in previous reports of the absence or low-level of global mtDNA methylation (Supplemental Table S1). In addition, we have at least eight lines of evidence to support mtDNA methylation is not a by-product of technical phenomena (Supplemental Information).

On the human mitochondria genome (16569 bp), the L strand C sites (5181) are more than twofold the number of C sites on the H strand (2169). Among 7350 C sites, only 435 C sites are CpGs. The CpG dinucleotide is pervasively underrepresented in all animal mitochondria compared with the nuclear genome. This may partially explain why mtDNA methylation mainly occurs on the L strand and on CpH sites. In addition, because 12 of 13 protein-coding genes use L strand as template, the regulation of the L strand by methylation may relate to the mitochondria function through mtDNA gene expression regulation.

The finding of L strand–biased methylation was consistent with a report examining several mtDNA loci (Bellizzi et al. 2013) and was confirmed in a hairpin bisulfite sequencing technique reanalysis (Supplemental Fig. S4C), which produces identical read coverage on the H and L strands to eliminate the possibility of bias from unequal strand read coverage. Further confirming this phenomenon, similar L strand methylation bias is observed using a non-bisulfite method, digestion with a methyl CC cutting restriction enzyme FspEI, followed by sequencing (Fig. 2B).

We observed substantial CpH methylation in the mitochondrial genome. Non-CpG methylation is rare in most tissues, but substantial in brain and embryonic stem cells (ESCs), where it is established and maintained by DNMT3A. Consistently we found that all mtDNA peak regions are non-CpGs, and KO of *DNMT3A* abolished a specific CpH peak in the *MT-ND2* gene, whereas OE of *DNMT3A* increased global mtDNA methylation levels at CpGs and non-CpGs (Fig. 4C).

A periodic methylation distance of DNMT3A is found in nuclear CpG islands (CGI) (Jia et al. 2007), which molecular modeling shows to be caused by spacing of active sites within tetrameric DNMT3A–DNMT3L complexes separated by about one DNA helical turn. However, the mitochondria genome does not have CGI, and we did not observe the 8- to 10-bp periodicity in mtDNA methylation patterning. It might be less obvious of a pattern in mtDNA if DNMT3A accesses target sites in mtDNA via different DNMT3A complexes or from different DNA context.

Within individual samples, gene expression and methylation are anti-correlated on both strands (Fig. 3C). However, in general, mtDNA methylation and transcription level both decreased in old human brain, suggesting other cofounding factors might affect mtDNA methylation and expression during aging.

In addition to the negative associated mtDNA methylation with mtDNA transcript level, when comparing *DNMT3A* OE versus control cells, we found that an increase in L-strand gene 5′ end methylation, in particular, caused a decrease in the processing of individual genes from the polycistronic transcript, which uses L strand as the transcription template. The transcript using H strand as template contains only one coding gene and thus needs no individualized control on its processing over other genes in the same transcript, which perhaps explains the low H strand methylation and implicates non-CpG mtDNA methylation as a transcription-coupled event.

Although we observed significant negative correlation/association of mtDNA methylation with mitochondrial gene expression, from our analysis, we could not rule out the nuclear effects on mitochondrial DNA methylation and gene expression. However, van der Wijst et al. (2017) have concluded direct causal effects of mtDNA methylation (through mitochondria targeting of bacterial or viral DNA methyltransferases) in the GpC, but not CpG context on mitochondrial gene expression, which supports our conclusion (Supplemental Information).

We noted there is residual mtDNA methylation detected in TKO mESCs, but the strand bias was completely abolished (Supplemental Fig. S6L). Because we also observed a similar low-level residual methylation also remained in nuclear DNA (Supplemental Fig. S6P), it is unclear whether such residual methylation caused by the noise of the BS-seq technique or other DNA methyltransferases yet to be identified for DNA methylation (Bellizzi et al. 2013). In either case, this further indicates that the strand-biased methylation maps represent true methylation patterns.

Our mapping mtDNA methylation across the mitochondrial genome revealed unprecedented insights into the mystery of individualized mitochondria gene expression control from a polycistronic transcription and opens up a new dimension of epigenetic regulation and a blueprint for future investigation of other tissues or other contexts, such as in metabolic reprogramming, dysfunction, and diseases, in which DNA methylation–regulated mitochondrial gene expression may regulate epigenetic and metabolic states.

## Methods

### Human samples

Caucasian American frontal cortex (PFC) samples were obtained from the National Institute of Child Health and Human Development (NICHD) Brain and Tissue Bank for Development Disorders and the Rush Religious Orders Study, Rush University Medical Center. Han Chinese frontal cortex samples were obtained from the Wuhan Brain Bank. All samples were obtained from individuals who had given written consent and were classified as “normal” and having death from causes unrelated to the tissue type being studied (for complete sample information, see Supplemental Table S3). RNA integrity of all human PFC samples was measured with an Agilent 2100 Bioanalyzer and the RNA Nano LabChip, and only individual samples with a RIN score greater than 5 were chosen for inclusion. Five to nine individuals per group within a ∼10 yr age bracket were pooled into groups (Supplemental Table S3). For this, equal amounts of each individual sample in the group were homogenized by freeze-fracturing frozen samples at −80°C using a homemade tissue pulverizer.

### Cell lines

Human cell lines HEK293T and A549 were grown in Dulbecco's Modified Eagle's Medium (DMEM; Gibco) with 10% fetal bovine serum (FBS; Gibco) and 1× Penicillin-Streptomycin-Glutamine (100 units/mL penicillin, 100 µg/mL streptomycin, 0.292 mg/mL L-glutamine; Gibco). Mouse E14 ESCs were grown on gelatin-coated plates with DMEM supplemented with 15% FBS, 1× Penicillin-Streptomycin, 1% nonessential amino acids (NEAA; Gibco), 100 µM β-mercaptoethanol (Millipore ES.007.E), 2 mM L-glutamine, and Leukemia inhibitory factor (LIF; 1000 units/mL; Millipore ESG1107). Mouse J1 wild-type and TKO mESCs were cultured in Glasgow Minimum Essential Medium (GMEM; Gibco) without feeders, supplemented with 15% FBS, 1× β-mercaptoethanol, 1000 units/mL LIF (Millipore ESG1107), 1× GlutaMAX, 1 mM sodium pyruvate, 1× NEAA, 50 units/mL Penicillin, 50 µg/mL Streptomycin (Gibco), 3 μM CHIR99021 (Selleck Chemicals S1263), and 1 μM PD0325901 (Selleck Chemicals S1036).

HEK293T *DNMT3A* KO cells were created by CRISPR-Cas9 targeting system using the 5′-GATGACGAGCCAGAGTACG-3′ sgRNA sequence described previously (Maeder et al. 2013) targeting Chromosome 2, 25248038-24248057 (reverse strand; hg38) that targets shared exons of the catalytic *DNMT3A* isoforms. Clones with homozygous loss of *DNMT3A* were selected, verified by sequencing, and confirmed for DNMT3A absence by western blotting.

J1 mESCs with *Dnmt1*/*Dnmt3a*/*Dnmt3b* triple KO were generated by Cre-mediated gene knockout as previously described (Tsumura et al. 2006).

### mtDNA extraction

#### Cell line mtDNA

Mitochondrial isolation followed the protocol of Clayton and Shadel (2014), with additional purification steps such that low-speed centrifugation is repeated one step beyond the last time there was a visible pellet of nucleus or was isolated as whole-cell DNA extracts.

#### PFC mtDNA

Extraction was performed using roughly 10–20 mg of pooled mitochondrial sample. Extraction of mtDNA was similar to the Clayton and Shadel purification method, performed using the mtDNA isolation kit (Abcam) with modifications to the manufacturers’ protocol: Dounce homogenization was performed for 25 strokes at 4°C, an additional low-speed spin at 700*g* was performed to improve the removal of cell debris and nuclei, 1 µL of Proteinase K (Fermentas) was added to the enzyme mix to degrade DNase, and a phenol-chloroform extraction was included before ethanol precipitation to increase the purity of the final DNA solution.

### Sodium bisulfite treatment

Bisulfite treatment of mtDNA was performed using the EpiTect Bisulfite Kit (QIAGEN). Two hundred nanograms (PFC samples) or 1 µg (cell line samples) of DNA was first converted using the “Bisulfite DNA conversion” steps of the manufacturers' protocol “Sodium Bisulfite Conversion of Unmethylated Cytosines in DNA,” with the optional additional cycle added to the conversion program (a final denaturation step for 5 min at 95°C, followed by 2 h at 60°C, and then hold at 20°C). To ensure the maximum recovery of DNA, cleanup was performed using the “Cleanup of bisulfite converted DNA” steps of the manufacturers’ protocol “Sodium Bisulfite Conversion of Unmethylated Cytosines in Small Amounts of Fragmented DNA” and using the optional carrier RNA during spin column cleanup. Contaminating RNA for PFC samples was removed with 1 µL RNase A (QIAGEN) for 10 min at 70°C with shaking in a total volume adjusted to 200 µL with ddH_2_O. RNase was subsequently removed by phenol-chloroform extraction, and DNA was isolated by isopropanol precipitation.

### Amplification of bisulfite-converted PFC mtDNA

Bisulfite-converted DNA was amplified using an isothermal Multiple Displacement Amplification (MDA) approach used in the EpiTect Whole Bisulfitome Kit (QIAGEN). MDA uses short random hexamer primers and a high-fidelity Phi 29 polymerase for amplification of large amounts of DNA in a nonspecific manner. All bisulfite-converted mtDNA was amplified according to the manufacturers’ protocol. Samples were cleaned using the QIAamp DNA Mini Kit (QIAGEN) and the supplemental manufacturers' protocol “Purification of REPLI-g amplified DNA using the ‘QIAamp DNA Mini Kit.’” DNA was concentrated with a final isopropanol precipitation step and resuspended in 50 µL EB buffer. Amplified converted mtDNA was quantified by 260 nm absorbance on a NanoDrop 2000 machine. Amplification yielded up to 7–8 µg of mtDNA (Supplemental Table S7).

### Background methylation on mtDNA

To further assess the accuracy of our mtDNA BS-seq technique, we checked the apparent background of our technique using unmethylated mtDNA prepared from four samples (M_25.6, W_27.7, M_46.8, W_50.5) using long-range PCR (LA Taq, TaKaRa) to amplify the complete mtDNA genome in two overlapping fragments, A and B (see below). According to the manufacturers’ recommendations, 50-µL PCR reactions were prepared, and reaction conditions were 5 min at 98°C, 40 cycles of denaturation for 30 sec at 98°C, annealing for 30 sec at 58°C, and extension for 10 min at 72°C, with a final extension for 5 min at 72°C. Products were agarose gel–purified on a 0.7% gel at 120V for 2 h in 1× TBE and gel extracted (Gel extraction kit, QIAGEN). Equimolar amounts of each fragment were mixed, and then BS-seq was performed as for purified mtDNA.

mtfullAF: AACCAAACCCCAAAGACACC
mtfullAR: GCCAATAATGACGTGAAGTCC
mtfullBF: TCCCACTCCTAAACACATCC
mtfullBR: TTTATGGGGTGATGTGAGCC

### MeDIP

Whole-cell DNA was extracted from HEK293T cells using the QIAamp DNA Mini Kit (QIAGEN), according to the manufacturers' protocol. The MeDIP protocol was based on Weber et al. (2005). Contaminating RNA was removed from both mtDNA and whole DNA with 1 µL RNase A (QIAGEN) for 30 min at 60°C with shaking in a total volume made up to 200 µL with ddH_2_O. RNase was subsequently removed by phenol-chloroform extraction, and DNA was precipitated with isopropanol. Two micrograms of DNA per MeDIP was sheared on a Covaris S220 focused-ultrasonicator using the Illumina 200 protocol (to yield 200 bp fragments) provided by the manufacturer. Shearing size was confirmed using an Agilent 2100 Bioanalyzer and the DNA 7500 LabChip. Sheared DNA was diluted to 455 µL in TE buffer and denatured at 95°C for 10 min, followed by ice cooling for 10 min. Then, 51 µL of 10× IP buffer (100 mM Na-Phosphate pH 7.0, 1.4 M NaCl, 0.5% Triton X-100) and 5 µL of either anti-5mC (Abcam ab10805) or anti-5hmC (Abcam ab106918) antibodies were added to the DNA and preincubated with overhead shaking for 2 h at 4°C. Twenty microliters Protein G Dynabeads (Life Technologies) were prewashed 2× with 800 µL 0.1% BSA in PBS for 5 min at RT with shaking. Beads were collected, resuspended in 40 µL of 1× IP buffer (10 mM Na-Phosphate pH 7.0, 0.14 M NaCl, 0.05% Triton X-100), and then added to the preincubated antibody-DNA mix. Tubes were incubated at 4°C overnight with overhead shaking, and then beads were washed 3× with 800 µL 1× IP buffer. Beads were resuspended in 250 µL of Proteinase K digestion buffer (50 mM Tris pH 8.0, 10 mM EDTA, 0.5% SDS) with 7 µL of Proteinase K (Fermentas) and incubated for 3 h at 50°C with shaking. DNA was then extracted using phenol-chloroform extraction followed by isopropanol precipitation (Weber et al. 2005).

### WGBS and MeDIP-seq

All DNA (bisulfite-treated whole-cell DNA, isolated mtDNA, amplified mtDNA, and MeDIP mtDNA) were analyzed by Illumina HiSeq 2000. Untreated mtDNA was prepared by extracting total cellular DNA using the DNA Mini Kit (QIAGEN) and amplifying mtDNA using the REPLI-g mitochondrial kit (QIAGEN) according to the manufacturers’ protocols. DNA Libraries were constructed using Illumina TruSeq DNA sample prep kit V2 by the Shanghai Institute for Biological Sciences core facility.

### WGBS and MeDIP-seq data processing

Human PFC WGBS data were uniquely mapped to the revised Cambridge human mtDNA Reference Genome (rCRS, NC_ 012920.1) using BSMAP (Xi and Li 2009) allowing two mismatches with methylation called using “methyratio.py.” C or G sites were excluded if the ratio of reads at that site showed a difference from the reference >5% to exclude the effect of a common C/T SNPs. There are 273 sequence differences from the reference in C/G sites across all human PFC samples, with around 36–64 sequence differences for each sample. Further, all duplicated reads with the same base composition and mapping position are removed. Although our BS-seq human PFC data were generated in two batches, the majority of the batch effects could be eliminated using ComBat (Supplemental Fig. S4E; Johnson et al. 2007).

Similar to human PFC samples, other in-house-generated and downloaded published human or mouse WGBS data were uniquely mapped to the rCRS (NC_012920.1) or ChrM of mm10 mouse reference genome using BSMAP (Xi and Li 2009) allowing two mismatches with methylation called using “methyratio.py.” Mouse mtDNA was compared to human mtDNA using the UCSC liftOver tool (Fig. 2A). MeDIP-seq data was uniquely mapped to the rCRS using Bowtie (v0.12.8) (Langmead 2010), and peaks were called using HOMER (http://homer.ucsd.edu/homer/index.html; Heinz et al. 2010) with customized parameters (-F 2 -L 2).

### RNA sequencing and data processing

RNA was extracted from all groups, except groups C_87.1 and W_27.7, W_37.3, W_50.5, and W_59.2 for which there was insufficient tissue. One hundred milligrams of tissue was lysed in TRIzol (Life Technologies) using a TissueRuptor (QIAGEN) to extract total RNA. RNA-seq library construction and HiSeq were performed by Berry Genomics. Illumina HiSeq was used to generate 50- and 100-bp single-end sequencing data. Reads were uniquely mapped to the rCRS (NC_012920.1) using Bowtie (v0.12.8) (Langmead 2010) as described above. MITOMAP (Brandon et al. 2005) -annotated mitochondrial gene expression was quantified by per million mapped reads per kilo base (rpkm) using a custom script (Supplemental Code). Here, mapped reads refers to reads mapped to RefSeq human genome hg19 using TopHat (v1.4.1) (Trapnell et al. 2009). Expression values were corrected for batch effect using ComBat (Johnson et al. 2007). A similar processing strategy was applied to other in-house-generated or downloaded published human or mouse RNA-seq data, except for batch effect correction.

### Immunofluorescence

HEK293T and A549 cells were cultured overnight on coverslips, stained with MitoTracker Orange at a final concentration of 300 nM for 20 min, then washed and fixed with 4% paraformaldehyde in PBS. Cells were blocked and permeabilized in PBS with 5% FBS, 1% BSA, and 0.3% Triton X-100 for 60 min, then incubated overnight at 4°C with gentle shaking, with antibodies diluted in PBS/1% BSA/0.3% Triton X-100, then washed in PBS, incubated with secondary antibodies for 60 min at room temperature, counterstained with DAPI, and imaged with confocal microscopy (Zeiss Axio Observer). Antibodies were used at the following dilutions: anti-HA (Cell Signaling Technologies, C29F4), 1:1600, anti-DNMT3A (Abcam, ab2850), 1:500. Cells were imaged in *Z*-stacks and analyzed with ImageJ.

### Mitochondria fractionation for western blot

Mitochondria were either isolated by a commercial Mitochondria Isolation kit ( 89874; Thermo Fisher Scientific) and followed manufacturer's instructions to separate mitochondria and cytoplasm or isolated as described above for mtDNA isolation in cell lines, with the following modifications: no DNase or RNase treatment, and after the final wash of the mitochondria pellet is performed, mitochondria were lysed. Mitochondria were homogenized with ice-cold RIPA lysis buffer, which contained 50 mM Tris-HCl pH 8.0, 150 mM NaCl, 0.1% sodium dodecyl sulphate (SDS), 0.5% Na-deoxycholate, 1% Nonidet P-40 (NP-40), and protease inhibitor tablet (Roche). After 5 min on ice, samples were sonicated with 15 sec on, 45 sec off, for three cycles (Bioruptor Plus), then the concentration of mitochondria protein was measured by Pierce BCA Protein Assay Kit (Thermo Fisher Scientific, 23225), and 10 µg of each sample was mixed with 5× SDS loading buffer and ddH_2_O for western blotting. Primary antibody treatment used DNMT3A antibodies (Abcam, ab2850 or Cell Signaling Technology, D23G1) was diluted 1:1000 in 5% w/v BSA, 1× TBST, and incubated membranes were put at 4°C with gentle shaking overnight.

### Oxygen respiration measurement

A549 and HEK293T cells were seeded onto a Seahorse XF24 24-well culture plate at 40,000 and 50,000 cells per well, respectively, and cultured overnight. Assay medium and drugs were prepared using Agilent Seahorse XF Base Medium (DMEM), with 10 mM glucose, 1 mM pyruvate, and 2 mM L-glutamine added before use. Respiration was perturbed using injections of oligomycin (1 µM) to inhibit ATP synthase; the uncoupler FCCP to stimulate maximal respiration; and a mix of rotenone and antimycin A (0.5 µM) to inhibit complex I and complex III and abolish mitochondrial respiration, all of which were provided in the Agilent Seahorse XF Cell Mito Stress Test Kit (Agilent) and dissolved in assay medium according to kit instructions. The Mito Stress Test assay was performed in a Seahorse XF24 Extracellular Flux Analyzer (Agilent) according to the manufacturer's instructions.

### DNA methylation–dependent and –independent restriction enzyme digestion

A total of 1 µg mtDNA isolated from wild-type and *DNMT3A*-KO HEK239T cells was digested in parallel using DNA methylation–dependent restriction enzyme FspEI (NEB, R0662S), which recognizes the C^m^C site (the second cytosine can be in the context of CG, CHG, or CHH). A similar experiment was performed by digesting the same amount of mtDNA with DNA methylation–independent restriction enzyme BstNI (NEB, R0168S), which recognizes the CCWGG (W = A or T) site. After digestion, the resulting mtDNA was subjected to agarose gel electrophoresis. DNA fragments <600 bp in the gel image were quantified with ImageJ.

### DNA preparation for HPLC-MS

Freshly isolated mouse cerebral cortex tissue was subjected to mtDNA, nuclear DNA, and genomic DNA isolation using mitochondrial DNA isolation kit (BioVision, K280) and QIAamp DNA Mini Kit (QIAGEN, 51304), respectively, according to the manufacturers’ protocols. To remove RNA contamination from DNA, 2 µL of freshly prepared RNase A/T1 (Thermo Fisher Scientific, EN0531, EN0541) mix was added and incubated for 1 h at 37°C. Nucleosides were derived from purified DNA (10 µg) by digestion with 2 units of Nuclease P1 (Sigma-Aldrich, N8630) overnight at 37°C, followed by treatment with 2 µL alkaline phosphatase (NEB, M0290) for 6 h at 37°C. The digestion product was centrifuged at 13,000 rpm for 15 min, and the supernatant was transferred into a clean microcentrifuge tube for further HPLC-MS analysis.

### Mitochondrial DNA methylation estimation using HPLC-MS data

Although both the 5mC and 5hmC levels observed in the mitochondrial were a bit less than nuclear DNA preparation, the ratio of 5hmC over 5mC was higher in mtDNA (22.18%) than nDNA (15.88%) (Fig. 5B). Because the preparation of mtDNA is unlikely to be completely devoid of nDNA, we assume the proportion of mtDNA in mitochondrial preparation is X, 5mC level on mtDNA is Y, 5hmC level of mtDNA is Z and the mtDNA in nuclear DNA component is negligible. Then we have (1−X) × n5mC_obs_ + X × Y = mt5mC_obs_, and (1−X) × n5hmC_obs_ + X × Z = mt5hmC_obs_, which according to our HPLC-MS measurements are (1−X) × 0.0644 + X × Y = 0.0432 and (1−X) × 0.0102 + X × Z = 0.0096) (Fig. 5B). Solving these two equations, we get *Z* = 0.0084 + *Y*/35.33. Although this equation gives no clue to the lower bound of 5mC on mtDNA, because of the higher than nuclear 5hmC to 5mC ratio mitochondria, this gives the lower bound of 5hmC on mtDNA as 0.84%, that is, even if assuming mtDNA does not have 5mC (*Y* = 0), we can obtain the minimum level of 5hmC that must exist on mtDNA as *Z*_min_ = 0.0084 (0.84%) from the equation above. While the maximum of *Z* and *Y* in the mouse brain are the 5hmC and 5mC levels detected (0.96% and 4.32%), it is assumed there is no contamination of nDNA in mtDNA component (Fig. 5B).

### Public data sets

Nine data sets from NCBI's Gene Expression Omnibus (GEO) with GSE numbers GSE48229, GSE49828, GSE56879 and GSE56697, GSE61457, GSE77003, GSE33722, GSE37578, and GSE29127 were downloaded and summarized in Supplemental Table S13 and processed in a similar way as described above.

## Data access

All raw and processed sequencing data generated in this study have been submitted to the NCBI Gene Expression Omnibus (GEO; https://www.ncbi.nlm.nih.gov/geo/) under accession number GSE133965. Essential codes to reproduce our result and test data are available as Supplemental Code.

## Supplementary Material

Supplemental Material
