# Supplementary material for: The strand-biased mitochondrial DNA methylome and its regulation by DNMT3A
Source: Genome Res. 2019 Oct;29(10):1622–34. doi: 10.1101/gr.234021.117 (PMC6771398; doi:10.1101/gr.234021.117)
Supplement: Supplemental Material [file supp_29_10_1622__index.html]

The strand-biased mitochondrial DNA methylome and its regulation by DNMT3A — Supplemental Material 

# The strand-biased mitochondrial DNA methylome and its regulation by DNMT3A

## Supplemental Material

- Supplemental\_Table\_S1.xlsx
- Supplemental\_Table\_S3.xlsx
- Supplemental\_Table\_S4.xlsx
- Supplemental\_Table\_S5.xlsx
- Supplemental\_Table\_S6.xlsx
- Supplemental\_Table\_S8.xlsx
- Supplemental\_Table\_S10.xlsx
- Supplemental\_Table\_S12.xlsx
- Supplemental\_Table\_S13.xlsx
- Supplemental\_Materials.docx
- Supplemental\_Code.zip
- Supplemental\_Figures\_.pdf
