# Supplementary material for: The strand-biased mitochondrial DNA methylome and its regulation by DNMT3A
Source: Genome Res. 2019 Oct;29(10):1622–34. doi: 10.1101/gr.234021.117 (PMC6771398; doi:10.1101/gr.234021.117)
Supplement: Supplemental Material [file supp_gr.234021.117_Supplemental_Materials_.docx]

**Supplemental Materials of**

**The strand-biased mitochondrial DNA methylome and its regulation by *DNMT3A***

Xiaoyang Dou^1,2,*^, Jerome D Boyd-Kirkup^1,*^, Joseph McDermott^1,*^, Xiaoli Zhang^1,3*^, Fang Li^1^, Bowen Rong^4^, Rui Zhang^1^, Bisi Miao^4^, Peilin Chen^5^, Hao Cheng^1^, Jianhuang Xue^6^, David Bennett^7^, Jiemin Wong^5^, Fei Lan^4^ and Jing-Dong J Han^1,3#^

^1^ Key Laboratory of Computational Biology, CAS Center for Excellence in Molecular Cell Science, Collaborative Innovation Center for Genetics and Developmental Biology, Chinese Academy of Sciences-Max Planck Partner Institute for Computational Biology, Shanghai Institutes for Biological Sciences, Chinese Academy of Sciences, 320 Yue Yang Road, Shanghai, 200031, China

^2^ University of Chinese Academy of Sciences, Beijing 100049, China

^3^ Peking-Tsinghua Center for Life Sciences, Academy for Advanced Interdisciplinary Studies, Center for Quantitative Biology (CQB), Peking University, Beijing 100871, China

^4^ Liver Cancer Institute, Zhongshan Hospital, Fudan University, Key Laboratory of Carcinogenesis and Cancer Invasion, Ministry of Education, Key Laboratory of Epigenetics, Shanghai Ministry of Education, and Institutes of Biomedical Sciences, Fudan University, Shanghai, 200032, China

^5^ Shanghai Key Laboratory of Regulatory Biology, Institute of Biomedical Sciences and School of Life Sciences, East China Normal University, Shanghai 200241, China

^6^ The State Key Laboratory of Molecular Biology, Institute of Biochemistry and Cell Biology, Chinese Academy of Sciences, Shanghai 200031, China

^7^ Rush Alzheimer's Disease Center, Rush University Medical Center, Chicago, IL, USA.

^*^ Co-first author

# Correspondence: [jackie.han@pku.edu.cn](mailto:jackie.han@pku.edu.cn)

**Supplemental Information**

**Evidence supporting the existence of high L-strand methylation**

The following five lines of evidence support that the observed high L-strand methylation is not due to special mtDNA secondary structure as has been assumed ([Liu et al. 2016](#_ENREF_5); [Mechta et al. 2017](#_ENREF_8); [Owa et al. 2018](#_ENREF_9)) or as a consequence of read coverage bias. 1) Where there are strand-symmetric CpGs, methylation is still biased to L strand, arguing against local secondary structure of double stranded mtDNA causing bisulfite resistance as being the cause of detection. 2) A strand biased mtDNA methylation pattern is similarly observed in published zebrafish and mouse development DNA methylation data, where sonication-linearization was performed before bisulfite treatment ([Jiang et al. 2013](#_ENREF_4); [Zhao et al. 2014](#_ENREF_13)); the observed strand-biased coverage and methylation of mtDNA is thus not dependent on whether mtDNA is circular or linear at the time of bisulfite treatment. 3) PGCs, having the same mtDNA, experimental treatment and absence of detected methylation, serve as a biological control for any artifact during BS-seq. 4) In hairpin bisulfite sequencing, the two strands of DNA fragments form a single amplicon with ligation of a hairpin adaptor during PCR amplification, resulting in identical mapped read coverage on the H and L strands, and can thus be used examine if the L strand high methylation is a result of lower mapping ratio compared to H strand. By re-analyzing two WGBS datasets prepared using hairpin bisulfite sequencing of mESCs ([Zhao et al. 2014](#_ENREF_13)), we still observed significantly higher methylation on the L strand (Supplemental Fig. 4C). 5) In addition we observed L strand-biased mtDNA methylation in mouse frontal cortex using the DNA methylation-dependent restriction enzyme FspEI-seq (a non-bisulfite based method) (Fig. 2B) ([Xie et al. 2012](#_ENREF_12)). These results show that strand-biased methylation is not dependent on unequal read mapping or coverage between the two strands, or due to inefficient bisulfite conversion.

**Explanation of mtDNA methylome following the pattern of nuclear methylome during early embryogenesis**

Nuclear remodeling is known to occur via a complex process involving many chromatin proteins including STELLA, UHRF1, DNMT1, DNMT3 and TET proteins. All of these proteins act within the chromatin context. Even though mtDNA has no canonical chromatin components, it was reported that *TFAM* can serve as the main (and possibly sole) mtDNA-coating protein (Ekstrand et al., 2004, Takamatsu et al., 2002) in higher concentrations and thus forming the bacterial-like DNA-protein structure, the nucleoid (Iborra et al., 2004, Kukat et al., 2015, Legros et al., 2004). Previous studies have demonstrated coordination between transcriptional regulation between the nucleus and mitochondria. For example, some modulators of [chromatin structure](https://www.sciencedirect.com/topics/biochemistry-genetics-and-molecular-biology/chromatin-structure) (mostly without mitochondria import signal), such as *KAT8* (Chatterjee et al., 2016), [*STAT3*](https://www.sciencedirect.com/topics/biochemistry-genetics-and-molecular-biology/stat3) (Macias et al., 2014), and *SIRT1* (Aquilano et al., 2010), as well as other known regulators of nuclear gene transcription (Blumberg et al., 2014, She et al., 2011) can import into the mitochondria and bind the mtDNA, raising the possibility that mtDNA higher-order organization is more structured and hence more regulated than once thought ([ShaniMarom](#!) et al., 2019). It is currently unstudied how DNMTs coordinate with other regulatory factors during development in mitochondria, but without similar chromatin contexts the coordination between DNMTs and other factors during developmental methylation are not expected to be identical.

**Potential effects of nucleus on regulating mtDNA methylome**

We tested known nuclear encoded mtDNA transcription factors to exclude the potential indirect effects from nuclear factors that could enter mitochondria to affect mitochondrial-gene expression. However, we cannot totally exclude the effects from the nucleus, as other unknown crosstalk mechanisms between nuclear DNA and mtDNA may exist.

According to Barres et al.’s report, *DNMT3B*, but not *DNMT1* or *DNMT3A*, prevented palmitate-induced non-CpG methylation of *PPARGC1A* (Romain Barres et al, 2009). In addition, we did not observe expression level changes of *PPARGC1A* after *DNMT3A* KO in HEK293T cells, but it was decreased after *DNMT3A* OE in A549 cells.

Sun *et al.* reported similar mtDNA methylation as we reported, and they also demonstrated the anti-correlation between mtDNA methylation and transcription (Xin Sun et al, 2018). In addition, their DNA samples were randomly fragmented to linearize mtDNA so as to overcome the overestimation of DNA methylation caused by the circular and supercoiling structure of mtDNA, which fully supports our observations. Unlike their finding, we found the mtDNA methylation occurs in a strand biased fashion and can be partially regulated by *DNMT3A*.

In van der Wijst et al.’s, study, they only focused on the D-loop region and mitochondrial *COX2* gene in three to four different cancer cell lines (HeLa, HCT116, SKOV3 and C33A), and found CpG and CpH level is low at these regions, then they expressed mitochondria-targeted DNA methyltransferases for CpG and GpC and confirmed the role of GpC on regulating mtDNA methylation. However, their results cannot rule out 1) the role of CpH on regulating mitochondrial gene expression, 2) existence of mtDNA methylation on the untested regions and 3) their potential roles in regulating mitochondrial function in other cells or tissues, as we observed in our study. However, the potential role of GpC methylation (which is part of GCH) they demonstrated supports our findings.

**Evidence to support mtDNA methylation is not a technical**

Altogether we have at least 8 lines of evidence to support mtDNA methylation is not a technical: 1) We used alternate approaches in BS-seq–with numerous variations in protocol tested – and non-bisulfite-based MeDIP and its corresponding input, and observed consistent peaks. The major bias in MeDIP-seq comes from high affinity for CG ([Down et al. 2008](#_ENREF_2)), which is not a bias in BS-seq, and in addition most mtDNA methylation peaks were non-CG. 2) We found that the methylation is biased toward L strand even at symmetrical CGs, where the mirroring Cs of the H strand are still lowly methylated compared with L, suggesting local secondary structure of a double strand DNA cannot be the cause of detected L strand high methylation (Fig. 2F). Additionally, using a well-controlled oocyte sonication-linearized BS-seq data ([Zhao et al. 2014](#_ENREF_13)), we still detected strong L strand specific methylation peaks that are similar to other data. These argue against these highly methylated peaks being an artifact of secondary structure-dependent inefficient bisulfite conversion. 3) We found FspEI methyl-C-dependent restriction enzyme digestion was partially dependent on DNMT3A, as mtDNA digestion was reduced in DNMT3A KO compared to wild type (Supplemental Fig. 6H). We also analyzed another dataset that used methyl-C-sensitive digestion followed by deep sequencing ([Xie et al. 2012](#_ENREF_12)). This showed presence of mtDNA methylation and consistently also showed higher L-strand specific methylation (1.9 fold compare with H strand; Fig. 2B right panels). 4) We used PCR generated negative mtDNA controls to address the reported effect of topology on resistance to bisulfite conversion ([Owa et al. 2018](#_ENREF_9)). In contrast to a similar control used by Owa et al, we found very low false-positive signal, perhaps due to their targeted focus on CpGs in a small region while we more fully evaluated false-positives by examining every C in the mitochondrial genome (Fig. 2C tracks labeled with "Neg Ctrl"). In contrast, in both partly-circular (at the time of bisulfite treatment) and linearized mtDNA samples, we observed high L strand methylation. 5) We validated the existence of methylation through its dependency on DNMTs. If the secondary structure of mtDNA prevented bisulfite conversion, detected methylation is not expected to be increased or decreased when DNMT3A is over- or underexpressed, or for methylation peaks and strand bias to completely disappear in Dnmt1, Dnmt3a and Dnmt3b TKO. 6) Random artifacts are unlikely to coincidentally generate biological significance: conserved methylation peaks across samples and tissues, and across different technologies; age- and development-dependent changes in methylation levels, dependency on DNMTs, correlation with mutation rate, matching functional effects on mitochondrial function and mitochondrial transcript maturation rate. 7) Previous analyses that failed to observe mtDNA methylation ([Maekawa et al. 2004](#_ENREF_6); [Hong et al. 2013](#_ENREF_3); [Matsuda et al. 2018](#_ENREF_7); [Owa et al. 2018](#_ENREF_9)) did not utilize strand-specific mapping of mtDNA methylation or focused only on CpGs (reflected in Table S1), thus overlooking high L-strand non-CpG methylation, which are consequently false-negatives in those analyses. 8) Using HPLC-MS, we identified higher 5hmC to 5mC ratio in mouse brain mtDNA, which is different from nDNA, although both levels are lower than nDNA.

**Supplemental Methods**

**Analysis of mtDNA copy number and mitochondrial gene expression by qPCR**

Relative mtDNA copy number was calculated using qPCR. Whole DNA was first extracted, then qPCR with 25 ng of DNA per reaction was performed using the SYBR Premix Ex Taq II kit (Takara) on an Agilent Technologies Stratagene MX3000P machine using the following program: Initial denaturation step for 30 s at 95^o^C, then 40 cycles of 5 s at 95 ^o^C and 20 s at 60 ^o^C, with a final cycle of 1 s at 95^o^C, 30 s at 65^o^C and 30 s at 95^o^C. qPCR of the mtDNA gene *ND4* and the nuclear housekeeping gene *APP* were carried out in triplicate (primers are listed in Table S8). Relative mtDNA copy number was calculated by comparing *ND4* to *APP* using the ΔΔCt method.

Isolation of mtDNA from PFC for WGBS was performed as above after comparison to the following methods. A whole genomic DNA extraction for PFC samples using the DNeasy Blood and Tissue Kit (QIAgen) had a 270-fold enrichment of nuclear DNA to that of mtDNA (Table S2), thus was not used for WGBS. With cultured cells, we compared the enrichment of the above optimized technique to other published and commercially available approaches, namely; the miniprep kit approach used by Quispe-Tintaya et al. ([Quispe-Tintaya et al. 2013](#_ENREF_11)), the QIAgen QProteome Mitochondria Isolation kit, and the Thermo Fisher Scientific Mitochondria Isolation Kit for Cultured Cells. For kits, after mitochondria isolation, mtDNA was extracted with Proteinase K treatment and phenol-chloroform purification, as necessary. In addition, for both kits, several alternative manufacturers protocols were used, namely the dounce homogenization approach and needle based lysis approach of the QProteome kit, the dounce homogenization approach and the reagent based lysis approach of the Thermo kit, as well as both it's “fast” and “slow” protocols for dounce and reagent protocols. Comparison of mtDNA enrichment was measured using qPCR as described above. We used the above optimized mtDNA isolation techniques due to significantly outperforming the others in enrichment of mtDNA. Estimating mtDNA constitutes 2% of total DNA, mtDNA represented over 99.9% of DNA in the isolated mtDNA from the PFC, and with cell lines nuclear DNA was nearly completely non-detectable with qPCR.

**Bisulfite conversion efficiency**

To test bisulfite conversion efficiency several approaches were used. In PFC samples we used regions with stable and known DNA methylation states which are only present in nuclear DNA. Total DNA was extracted from 10 mg of 6 frontal cortex pools using the DNAeasy Blood and Tissue Kit (QIAgen) according to the manufacturers protocol, and then bisulfite treated (Method). PCR (described below) was performed across two known unmethylated regions in nuclear DNA, *ES1R* and *BCL2*, for 3 randomly chosen PFC groups as well as an Epitect Control complete converted unmethylated DNA (QIAgen), using primer sets obtained from methPrimerDB ([Pattyn et al. 2003](#_ENREF_10)) (Table S8). In cell lines and downloaded datasets, unmethylated lambda DNA spike-in controls showed 99% or greater conversion efficiency.

Step-down Hot-start PCR was performed using LA taq polymerase (Takara) in a PCR reaction mix as per the manufacturers recommendation, and the following program: Initial denaturation step for 5 min at 95^o^C, 3 cycles of denaturation for 30 s at 95^o^C, annealing for 30 s at 54^o^C, and extension for 30 s at 72^o^C, 4 cycles with annealing at 51^o^C, 5 cycles with annealing at 48^o^C, and 25 cycles with annealing at 45^o^C, and a final extension for 3 mins at 72^o^C. Products were agarose gel purified on a 1% gel at 100V for 45 min in 1× TBE and gel extracted (Gel extraction kit, QIAgen). Products were then TA cloned into the pMD18-T vector (Takara, cat no. D101A) according to the manufacturers protocol. Clones for each PCR product were sequenced by Sanger sequencing (Sangon, China) using vector specific sequencing primers. Sequences were analysed by BiQ Analyser for conversion efficiency ([Bock et al. 2005](#_ENREF_1)). For both *ES1R* and *BCL2*, conversion efficiencies for frontal cortex groups of greater than 99% were observed (Table S9).

**Evaluating the influence of “NUMTs”**

Small regions of mitochondrial sequence inserted into the nuclear genome are known as “NUMTs”. In order to evaluate whether reads that mapped to ChrM were derived from NUMTs region, we remapped ChrM reads, to the nuclear DNA. Non-ChrM unique reads (NUMTs overlapping) were then compared against ChrM unique reads (non-NUMTs overlapping) to determine if these were derived from NUMTs, or conversely from the mitochondria.

We found that around 40-46% of the reads could also be mapped to nuclear DNA, while the other 54-60% could not, indicating that at least these 54-60 % of our reads are unique to the mitochondrial genome (Table S10). Nevertheless, we find that both fractions have exactly the same strand biased properties as the unique mitochondria reads (one strand of a chromosome shows high methylation and low coverage, and the other vice-versa) (Table S4 c.f Table S6). The expected symmetry for nuclear DNA was confirmed in the published whole genome bisulfite sequencing datasets (WGBS) (mESC cells) and for our datasets by reduced representation bisulfite sequencing (RRBS; Supplemental Fig. 4A-C).

Meanwhile, all NUMTs with matching RNA-seq reads in our analysis do not include the promoter region of mtDNA and, therefore, for it to be theoretically possible for them to be transcribed from the nuclear DNA, must be located within an existing nuclear gene and under the control of that gene’s promoter. For the majority of NUMTs, they are in fact located within nuclear intergenetic regions (around 95%; Table S11). For those that are located within another gene (around 5%), if they are transcribed, there should be a similar frequency of reads mapping to the NUMT as the frequency of RNA-seq reads that map to the flanking regions (500 bp upstream and downstream) of the gene within which it is located. We observe that there is a huge difference between these numbers of reads (around 500-10,000 fold comparing the reads from possible NUMTs vs. flanking regions for the same nuclear gene), again suggesting that although some mitochondrial RNA-seq reads may map to both nuclear and mitochondrial genomes, they are in fact predominantly derived from the mitochondrial genome (Table S12).

We separated C sites on mtDNA into two categories: NUMTs and non-NUMTs C sites. NUMTs Cs are C sites covered by reads that can be mapped to BOTH nuclear DNA and mtDNA, and non-NUMTs Cs are C sites covered by reads that can be mapped ONLY to mtDNA when mapping with whole genome including nuclear and mtDNA as reference.

**HPLC-MS peak quantification**

Each peak was quantified by Thermo Fisher Scientific Xcalibur^TM^ software, and the area (AA) under C, 5hmC or 5mC peak were determined at molecular weights 228.09788，258.10845 and 242.11353, respectively. Then the absolute levels of C (C_abs_), 5hmC (5hmC_abs_) and 5mC (5mC_abs_) on total genomic DNA (gDNA), nuclear DNA (nDNA) and purified mtDNA, respectively, were then calculated according to the standard curves of the area under peaks for C, 5hmC and 5mC at 0.25uM, 0.5uM, 1uM, 5uM and 10uM. Finally, the relative levels of 5hmC and 5mC to total C on gDNA, nDNA and purified mtDNA in WT mouse brain were calculated as 5hmC_abs_/(C_abs+_5hmC_abs+_5mC_abs_) and 5mC = 5mC_abs_/(C_abs+_5hmC_abs+_5mC_abs_)), respectively.

**Supplemental Tables**

**Table S2. mtDNA enrichment after isolation.**

| Cell type | DNA preparation | DNA Yield([Maekawa et al. 2004](#_ENREF_6)) | Relative mtDNA copy number | Estimated mtDNA vs. total DNA^*^ |
| --- | --- | --- | --- | --- |
| Human Brain^†^ | DNeasy Blood and Tissue | 17.613 | 1.000 | 2.000% |
|  | mtDNA Isolation protocol | 1.444 | 270.000 | 99.930% |
|  |  |  |  |  |
| HeLa | DNeasy Blood and Tissue | 18.550 | 1.000 | 2.000% |
|  | mtDNA Isolation protocol | 3.895 | 90.510 | 99.405% |
|  | Qiagen needle | 4.895 | 2.514 | 11.425% |
|  | Qiagen dounce | 1.415 | 36.929 | 96.532% |
|  | Thermo fast reagent | 1.780 | 2.423 | 10.698% |
|  | Thermo fast dounce | 2.210 | 3.630 | 21.193% |
|  | Thermo slow dounce | 0.740 | 9.383 | 64.243% |
|  | Thermo slow reagent | 1.300 | 3.605 | 20.963% |
|  |  |  |  |  |
| Mouse ES cells | DNeasy Blood and Tissue | 7.040 | 1.000 | 2.000% |
|  | mtDNA Isolation protocol | 1.088 | 21.656 | 90.540% |
|  | Miniprep protocol | 0.270 | 13.423 | 78.620% |

* mtDNA content was estimated as 2% of total cellular DNA. Actual content will vary.

† A representative human prefrontal cortex sample was used for testing isolation.

**Table S7. Amplification yields.**

| Group ID | MDA yield (ng)^*^ |
| --- | --- |
| M_25.6 | 3570 |
| W_27.2 | 5920 |
| W_27.7 | 3315 |
| M_34.0 | 3100 |
| W_37.3 | 2730 |
| W_40.7 | 2180 |
| M_46.8 | 2230 |
| W_50.5 | 2040 |
| W_53.9 | 7698.6 |
| W_59.2 | 2075 |
| M_62.2 | 6010 |
| C_76.7 | 615 |
| C_87.1 | 4245 |
| C_90.2 | 8250 |

* Yield after multiple displacement amplification (MDA) of bisulfite converted mtDNA

**Table S9. Bisulfite conversion efficiencies.**

| Nuclear Gene* | Group ID | Conversion efficiency (%)^†^ |
| --- | --- | --- |
| *ES1R* | W_27.2 | 100 |
|  | W_53.9 | 100 |
|  | C_76.7 | 99 |
|  | Control | 100 |
| *BCL2* | W_27.2 | 99.5 |
|  | W_53.9 | 99 |
|  | C_90.2 | 99 |
|  | Control | 99 |

* Nuclear gene for which the amplicon used to calculate conversion is known to be unmethylated.

† Sodium Bisulfite treatment cytosine conversion efficiency, calculated using BiQ Analyzer.

**Table S11. RNA-seq read mapping to ChrM and their corresponding locations in the nuclear genome.**

|  | All ChrM mapping reads | | | Potential NUMTs | | | | | |
| --- | --- | --- | --- | --- | --- | --- | --- | --- | --- |
| Group ID |  |  |  |  |  |  |  |  |  |
|  | ChrM | Also nuclear | Ratio | Exon | Ratio | Intron | Ratio | Intergenic | Ratio |
| M_25.6 | 3023594 | 1510849 | 0.500 | 7555300 | 0.049 | 0 | 0.0000 | 146781000 | 0.95 |
| W_27.2 | 3024868 | 2337889 | 0.773 | 6517980 | 0.052 | 179559 | 0.0014 | 119285111 | 0.95 |
| M_34.0 | 3399106 | 1682037 | 0.495 | 6989578 | 0.041 | 0 | 0.0000 | 164522422 | 0.96 |
| W_40.7 | 2244117 | 1682315 | 0.750 | 3701000 | 0.041 | 228372 | 0.0025 | 86824878 | 0.96 |
| M_46.8 | 2252307 | 1106888 | 0.491 | 5398828 | 0.048 | 0 | 0.0000 | 107258372 | 0.95 |
| W_53.9 | 3174813 | 2436965 | 0.768 | 5736387 | 0.043 | 292954 | 0.0022 | 125891509 | 0.95 |
| M_62.2 | 2918299 | 1439657 | 0.493 | 7778504 | 0.053 | 0 | 0.0000 | 139265896 | 0.95 |
| C_76.7 | 1547781 | 1183818 | 0.765 | 2777163 | 0.044 | 114111 | 0.0018 | 60951426 | 0.95 |
| C_90.2 | 2174932 | 1587305 | 0.730 | 2785787 | 0.032 | 214157 | 0.0025 | 83151656 | 0.97 |

**Supplemental References**

Bock C, Reither S, Mikeska T, Paulsen M, Walter J, Lengauer T. 2005. BiQ Analyzer: visualization and quality control for DNA methylation data from bisulfite sequencing. *Bioinformatics* **21**: 4067-4068.

Down TA, Rakyan VK, Turner DJ, Flicek P, Li H, Kulesha E, Graf S, Johnson N, Herrero J, Tomazou EM et al. 2008. A Bayesian deconvolution strategy for immunoprecipitation-based DNA methylome analysis. *Nature biotechnology* **26**: 779-785.

Hong EE, Okitsu CY, Smith AD, Hsieh CL. 2013. Regionally specific and genome-wide analyses conclusively demonstrate the absence of CpG methylation in human mitochondrial DNA. *Mol Cell Biol* **33**: 2683-2690.

Jiang L, Zhang J, Wang JJ, Wang L, Zhang L, Li G, Yang X, Ma X, Sun X, Cai J et al. 2013. Sperm, but not oocyte, DNA methylome is inherited by zebrafish early embryos. *Cell* **153**: 773-784.

Liu B, Du Q, Chen L, Fu G, Li S, Fu L, Zhang X, Ma C, Bin C. 2016. CpG methylation patterns of human mitochondrial DNA. *Sci Rep* **6**: 23421.

Maekawa M, Taniguchi T, Higashi H, Sugimura H, Sugano K, Kanno T. 2004. Methylation of mitochondrial DNA is not a useful marker for cancer detection. *Clinical chemistry* **50**: 1480-1481.

Matsuda S, Yasukawa T, Sakaguchi Y, Ichiyanagi K, Unoki M, Gotoh K, Fukuda K, Sasaki H, Suzuki T, Kang D. 2018. Accurate estimation of 5-methylcytosine in mammalian mitochondrial DNA. *Sci Rep* **8**: 5801.

Mechta M, Ingerslev LR, Fabre O, Picard M, Barres R. 2017. Evidence Suggesting Absence of Mitochondrial DNA Methylation. *Front Genet* **8**: 166.

Owa C, Poulin M, Yan L, Shioda T. 2018. Technical adequacy of bisulfite sequencing and pyrosequencing for detection of mitochondrial DNA methylation: Sources and avoidance of false-positive detection. *PLoS One* **13**: e0192722.

Pattyn F, Speleman F, De Paepe A, Vandesompele J. 2003. RTPrimerDB: the real-time PCR primer and probe database. *Nucleic Acids Res* **31**: 122-123.

Quispe-Tintaya W, White RR, Popov VN, Vijg J, Maslov AY. 2013. Fast mitochondrial DNA isolation from mammalian cells for next-generation sequencing. *BioTechniques* **55**: 133-136.

Xie W, Barr CL, Kim A, Yue F, Lee AY, Eubanks J, Dempster EL, Ren B. 2012. Base-resolution analyses of sequence and parent-of-origin dependent DNA methylation in the mouse genome. *Cell* **148**: 816-831.

Zhao L, Sun MA, Li Z, Bai X, Yu M, Wang M, Liang L, Shao X, Arnovitz S, Wang Q et al. 2014. The dynamics of DNA methylation fidelity during mouse embryonic stem cell self-renewal and differentiation. *Genome Res* **24**: 1296-1307.
