# Supplementary material for: The strand-biased mitochondrial DNA methylome and its regulation by DNMT3A
Source: Genome Res. 2019 Oct;29(10):1622–34. doi: 10.1101/gr.234021.117 (PMC6771398; doi:10.1101/gr.234021.117)
Supplement: Supplemental Material [file supp_gr.234021.117_Supplemental_Figures_.pdf]

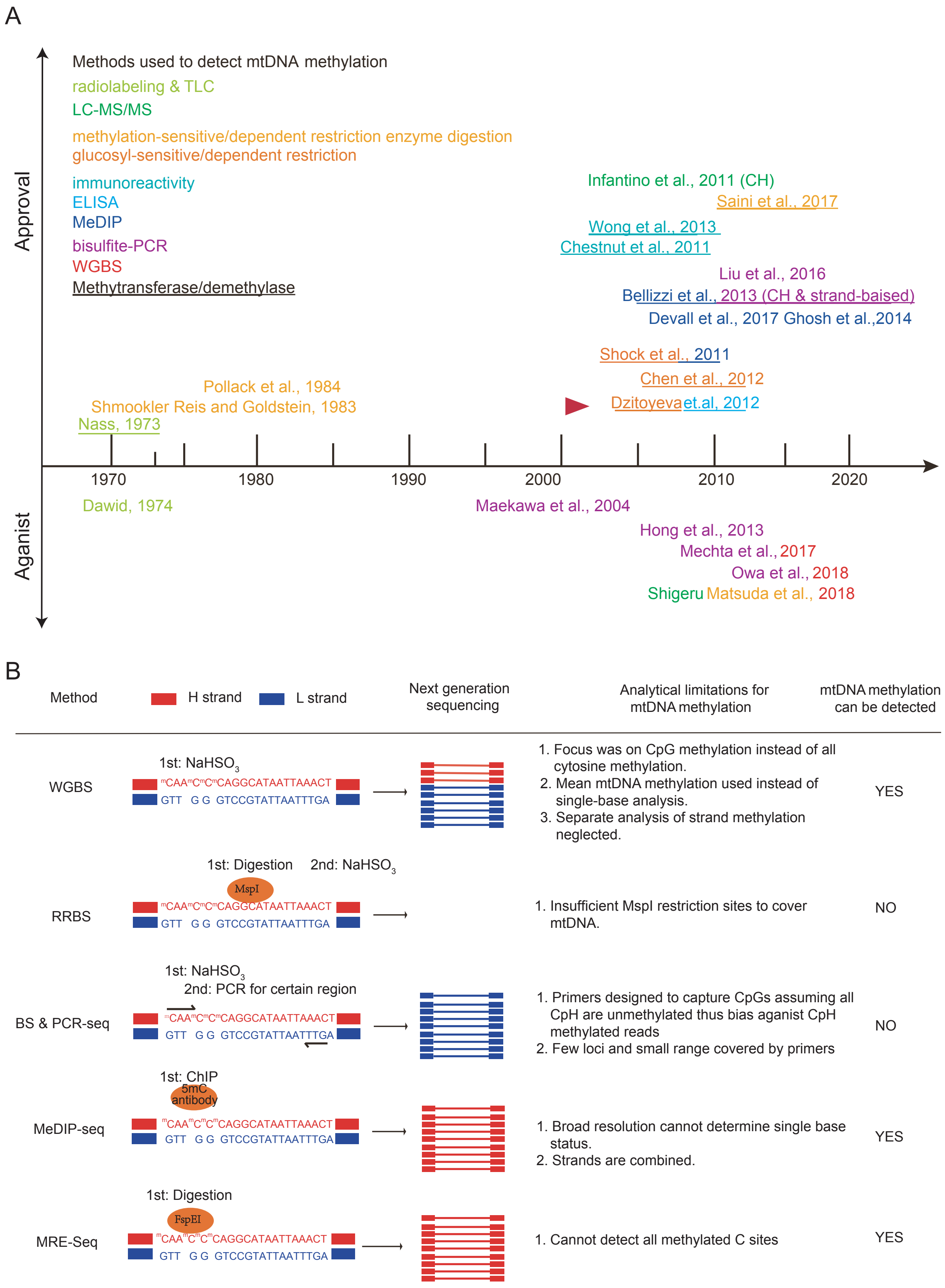
A
Approval
Aganist
Methods used to detect mtDNA methylation
radiolabeling & TLC
LC-MS/MS
methylation-sensitive/dependent restriction enzyme digestion
glucosyl-sensitive/dependent restriction
immunoreactivity
ELISA
MeDIP
bisulfite-PCR
WGBS
Methytransferase/demethylase
Infantino et al., 2011 (CH)
Saini et al., 2017
Wong et al., 2013
Chestnut et al., 2011
Liu et al., 2016
Bellizzi et al., 2013 (CH & strand-baised)
Devall et al., 2017 Ghosh et al.,2014
Shock et al., 2011
Chen et al., 2012
Dzitoyeva et.al, 2012
Pollack et al., 1984
Shmookler Reis and Goldstein, 1983
Nass, 1973
1970
1980
1990
2000
2010
2020
Dawid, 1974
Maekawa et al., 2004
Hong et al., 2013
Mechta et al., 2017
Owa et al., 2018
Shigeru Matsuda et al., 2018
B
Method
H strand
L strand
Next generation sequencing
Analytical limitations for mtDNA methylation
mtDNA methylation can be detected
WGBS
1st: NaHSO3
mCAAmCmCmCAGGCATAATTAAACT
GTT G G GTCCGTATTAATTTGA
1. Focus was on CpG methylation instead of all cytosine methylation.
2. Mean mtDNA methylation used instead of single-base analysis.
3. Separate analysis of strand methylation neglected.
YES
RRBS
1st: Digestion 2nd: NaHSO3
MspI
1. Insufficient MspI restriction sites to cover mtDNA.
NO
BS & PCR-seq
1st: NaHSO3
2nd: PCR for certain region
1. Primers designed to capture CpGs assuming all CpH are unmethylated thus bias aganist CpH methylated reads
2. Few loci and small range covered by primers
NO
MeDIP-seq
1st: ChIP
5mC antibody
1. Broad resolution cannot determine single base status.
2. Strands are combined.
YES
MRE-Seq
1st: Digestion
FspEI
1. Cannot detect all methylated C sites
YES

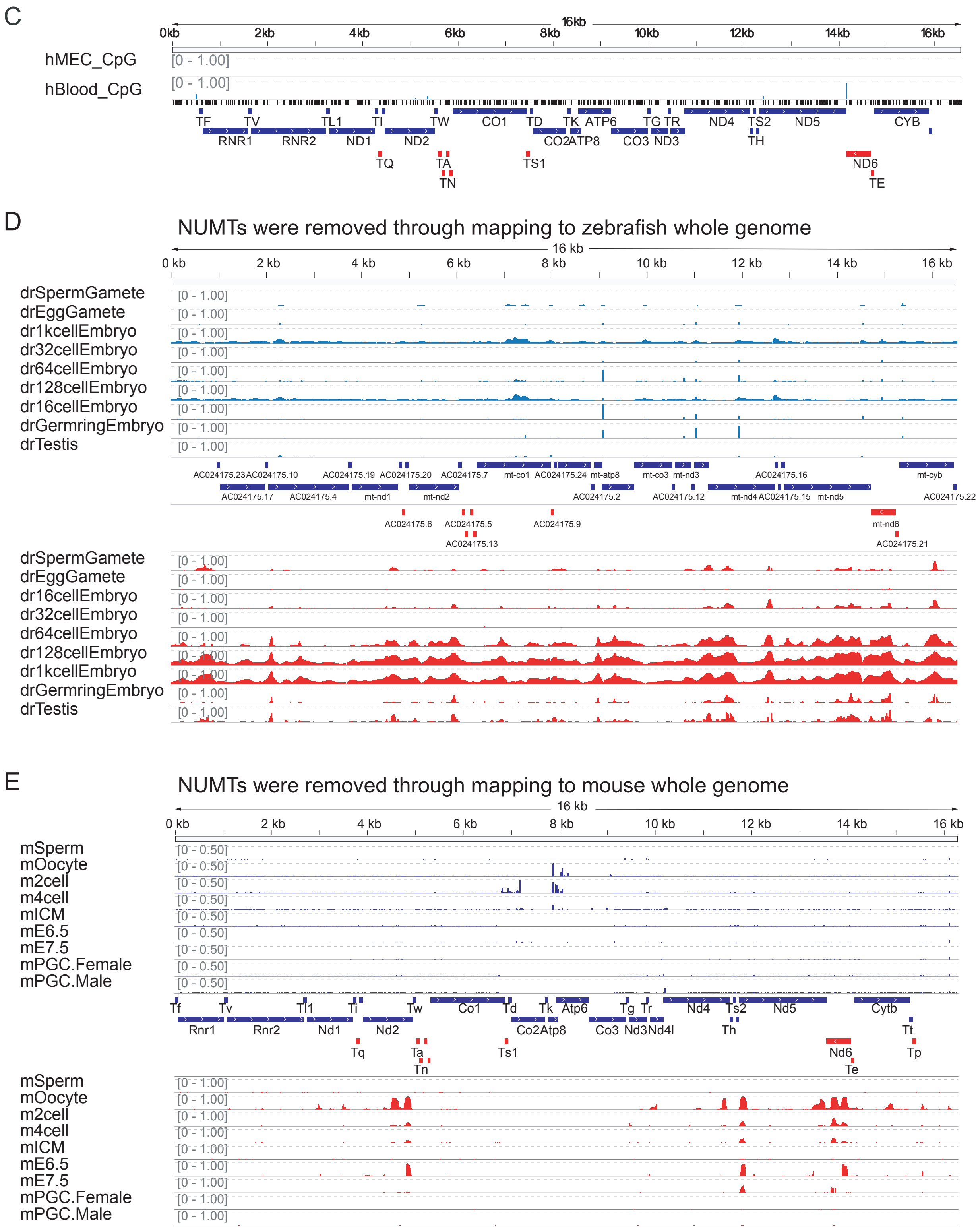

C
16kb
0kb
2kb
4kb
6kb
8kb
10kb
12kb
14kb
16kb
hMEC_CpG
[0 - 1.00]
hBlood_CpG
[0 - 1.00]
TF
TV
TL1
TI
TW
CO1
TD
TK
ATP6
TG
TR
ND4
TS2
ND5
CYB
RNR1
RNR2
ND1
ND2
CO2
ATP8
CO3
ND3
TH
TQ
TA
TS1
ND6
TN
TE
D
NUMTs were removed through mapping to zebrafish whole genome
16 kb
0 kb
2 kb
4 kb
6 kb
8 kb
10 kb
12 kb
14 kb
16 kb
drSpermGamete
drEggGamete
dr1kcellEmbryo
dr32cellEmbryo
dr64cellEmbryo
dr128cellEmbryo
dr16cellEmbryo
drGermringEmbryo
drTestis
[0 - 1.00]
AC024175.23
AC024175.10
AC024175.19
AC024175.20
AC024175.7
mt-co1
AC024175.24
mt-atp8
mt-co3
mt-nd3
AC024175.16
mt-cyb
AC024175.17
AC024175.4
mt-nd1
mt-nd2
AC024175.2
AC024175.12
mt-nd4
AC024175.15
mt-nd5
AC024175.22
AC024175.6
AC024175.5
AC024175.9
mt-nd6
AC024175.13
AC024175.21
drSpermGamete
drEggGamete
dr16cellEmbryo
dr32cellEmbryo
dr64cellEmbryo
dr128cellEmbryo
dr1kcellEmbryo
drGermringEmbryo
drTestis
[0 - 1.00]
E
NUMTs were removed through mapping to mouse whole genome
16 kb
0 kb
2 kb
4 kb
6 kb
8 kb
10 kb
12 kb
14 kb
16 kb
mSperm
mOocyte
m2cell
m4cell
mICM
mE6.5
mE7.5
mPGC.Female
mPGC.Male
[0 - 0.50]
Tf
Tv
Tl1
Ti
Tw
Co1
Td
Tk
Atp6
Tg
Tr
Nd4
Ts2
Nd5
Cytb
Rnr1
Rnr2
Nd1
Nd2
Co2
Atp8
Co3
Nd3
Nd4l
Th
Tt
Tq
Ta
Ts1
Nd6
Tp
Tn
Te
mSperm
mOocyte
m2cell
m4cell
mICM
mE6.5
mE7.5
mPGC.Female
mPGC.Male
[0 - 1.00]

Supplemental Fig. 1. WGBS of mtDNA quality control.

(A) Summary of literature evidence for presence or absence of mtDNA methylation.

(B) Analytical limitation of different methods in detecting mtDNA methylation.

(C) BS-seq methylation (WGBS) profiles of linearized mtDNA in human B cells (GSM922328 under GSE37578) and mammary epithelial cells (MEC, GSM721195 under GSE29127) on only CG sites.

(D-E) BS-seq (WGBS) methylation profiles of linearized mtDNA in zebrafish (D) as in Figure 1B, and mouse (E) as in Figure 1C embryonic development with NUMTs removed through mapping to whole genome.

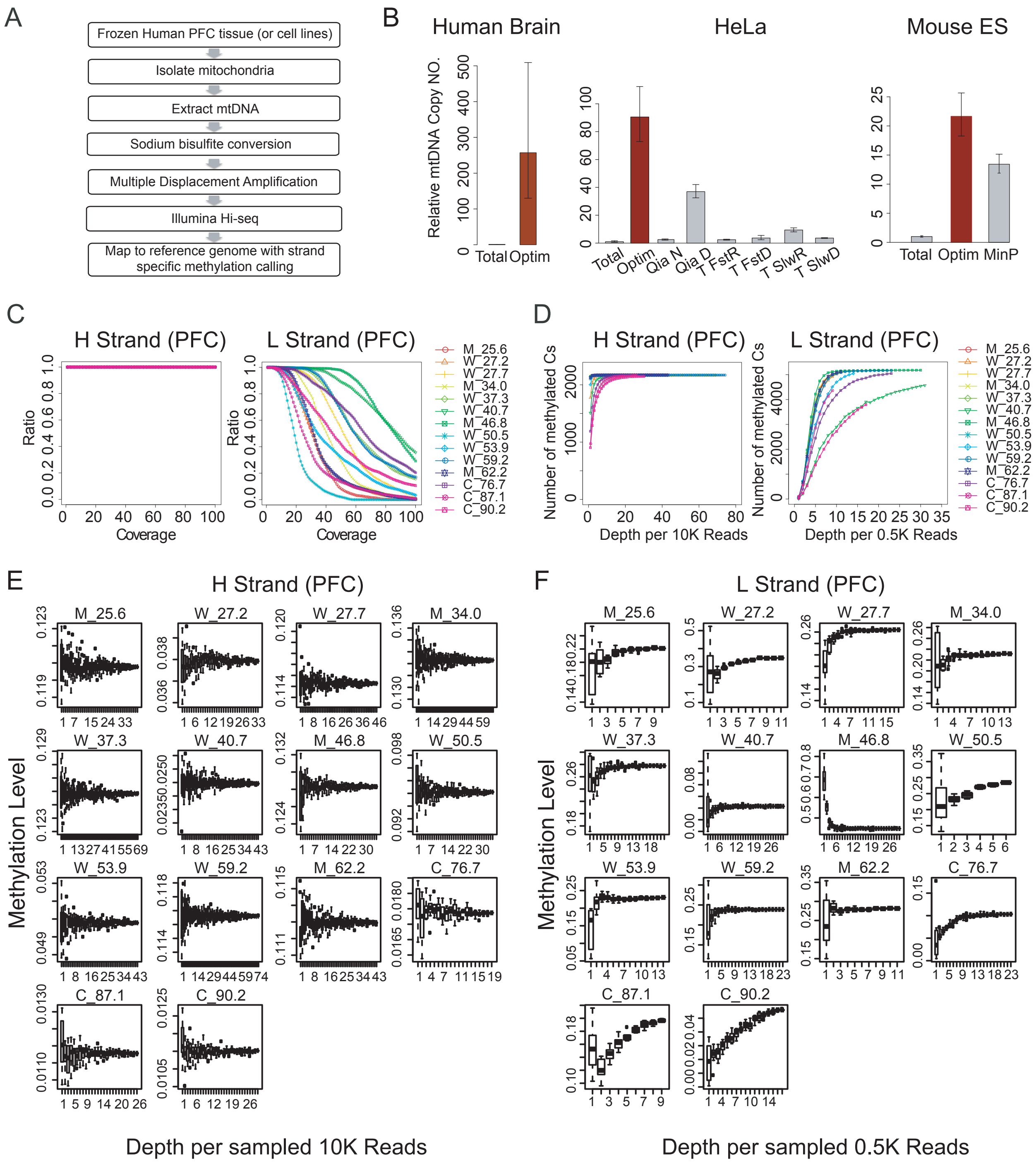
A
Frozen Human PFC tissue (or cell lines)
Isolate mitochondria
Extract mtDNA
Sodium bisulfite conversion
Multiple Displacement Amplification
Illumina Hi-seq
Map to reference genome with strand specific methylation calling
B
Human Brain
HeLa
Mouse ES
Relative mtDNA Copy NO.
Total Optim
Total Optim Qia N Qia D T FstR T FstD T SlwR T SlwD
Total Optim MinP
C
H Strand (PFC)
L Strand (PFC)
Ratio
Coverage
M_25.6
W_27.2
W_27.7
M_34.0
W_37.3
W_40.7
M_46.8
W_50.5
W_53.9
W_59.2
M_62.2
C_76.7
C_87.1
C_90.2
D
H Strand (PFC)
L Strand (PFC)
Number of methylated Cs
Depth per 10K Reads
Depth per 0.5K Reads
E
H Strand (PFC)
Methylation Level
Depth per sampled 10K Reads
F
L Strand (PFC)
Methylation Level
Depth per sampled 0.5K Reads

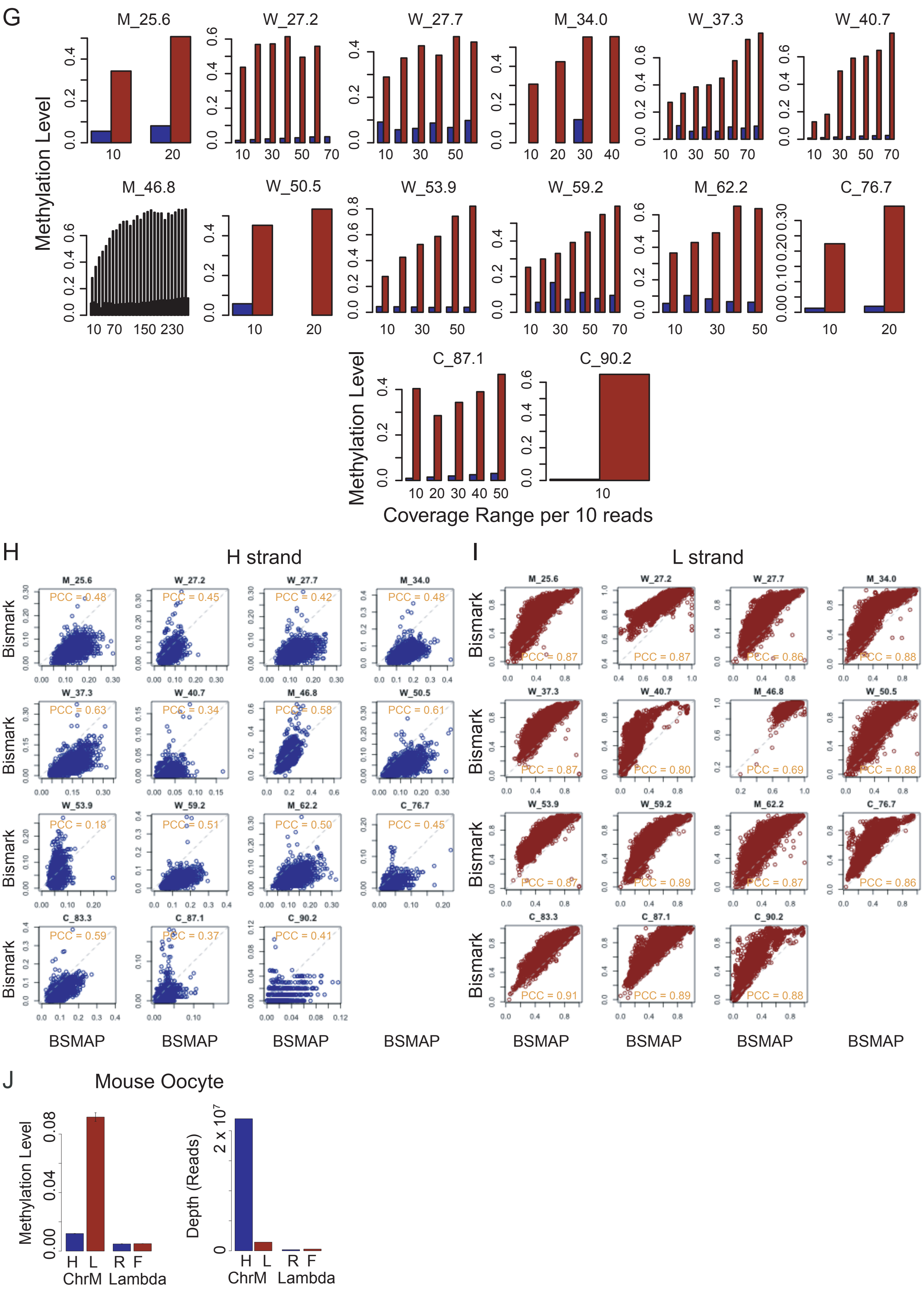

G
M_25.6
W_27.2
W_27.7
M_34.0
W_37.3
W_40.7
M_46.8
W_50.5
W_53.9
W_59.2
M_62.2
C_76.7
C_87.1
C_90.2
Methylation Level
Coverage Range per 10 reads
H
H strand
I
L strand
Bismark
BSMAP
PCC = 0.48
PCC = 0.45
PCC = 0.42
PCC = 0.48
PCC = 0.63
PCC = 0.34
PCC = 0.58
PCC = 0.61
PCC = 0.18
PCC = 0.51
PCC = 0.50
PCC = 0.45
PCC = 0.59
PCC = 0.37
PCC = 0.41
PCC = 0.87
PCC = 0.87
PCC = 0.86
PCC = 0.88
PCC = 0.87
PCC = 0.80
PCC = 0.69
PCC = 0.88
PCC = 0.87
PCC = 0.89
PCC = 0.87
PCC = 0.86
PCC = 0.91
PCC = 0.89
PCC = 0.88
J
Mouse Oocyte
Methylation Level
Depth (Reads)
H L R F
ChrM Lambda

Supplemental Fig. 2. WGBS of hPFC mtDNA quality control.

(A) The mtDNA enrichment and whole mtDNA BS-seq workflow.

(B) Relative mtDNA copy number enrichment using specified mtDNA isolation techniques in HeLa and mouse ES cells.

(C) BS-seq read coverage on mtDNA. Coverage (x-axis) is the number of reads that cover a certain position. Ratio (y-axis) is number of positions with certain coverage divided by length of mtDNA.

(D) Saturation plots for number of covered Cs by at least 10 reads, from cumulative random sampling of a subset of reads in 10,000 (10k) read blocks for H strand (left panel), or 5000 (0.5k) for L strand (right panel).

(E-F) Saturation plots for mean methylation level of Cs covered by at least 10 reads, from cumulative random sampling of a subset of reads in 10,000 (10k) read blocks for H strand (E), or 5000 (0.5k) for L strand (F).

(G) Methylation level of C on H and L strand with similar coverage.

(H-I) Correlation of mtDNA methylation called by BSMAP and Bismark on H (H) and L strand (I) separately, before any further filtering.

(J) Strand specific methylation level and sequence depth on ChrM, and non-strand specific methylation level and sequence depth on lambda DNA. Error bars represent SEM among C sites covered by at least ten reads.

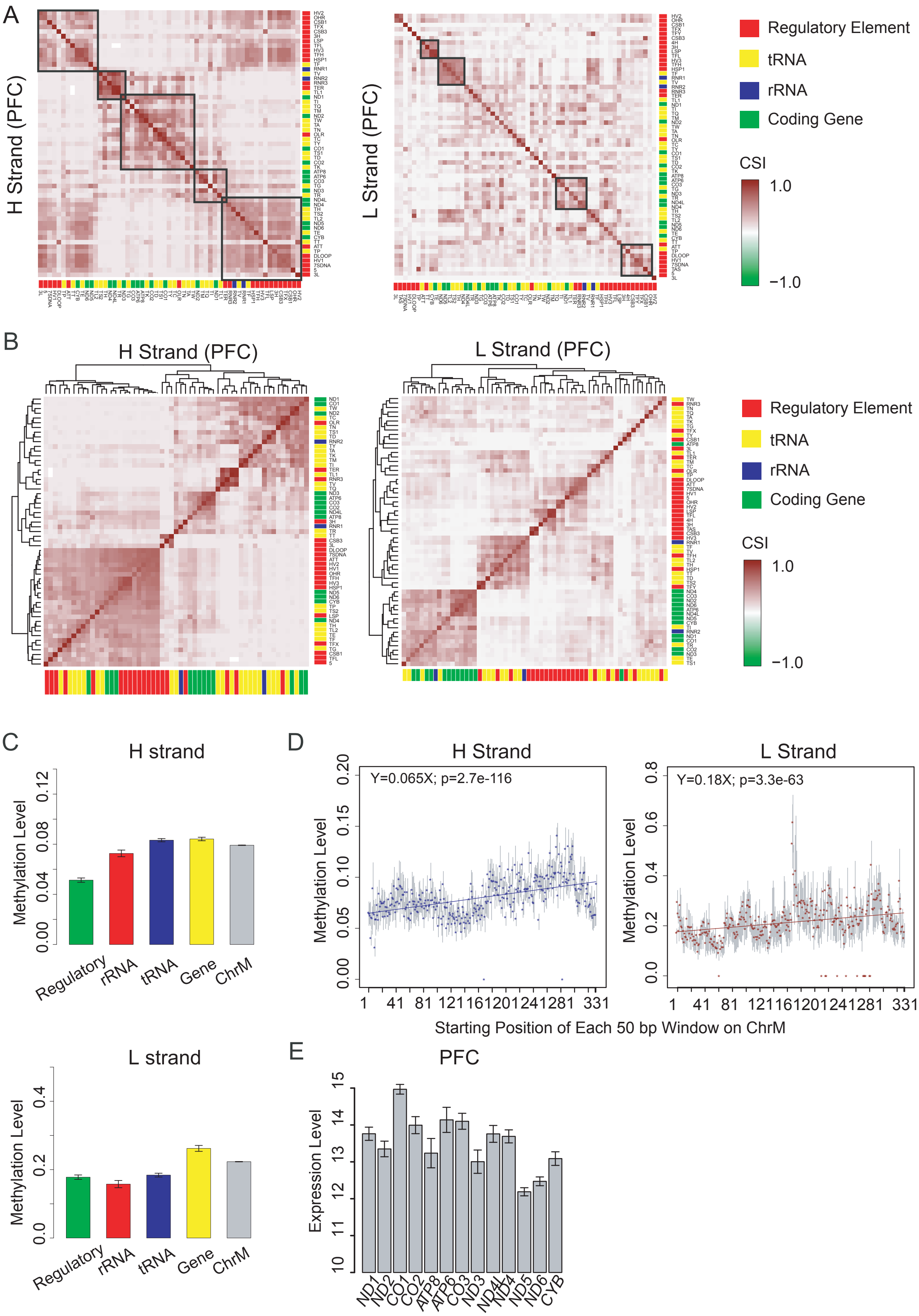
A
H Strand (PFC)
L Strand (PFC)
Regulatory Element
tRNA
rRNA
Coding Gene
CSI
1.0
−1.0
B
H Strand (PFC)
L Strand (PFC)
Regulatory Element
tRNA
rRNA
Coding Gene
CSI
1.0
−1.0
C
H strand
Methylation Level
Regulatory
rRNA
tRNA
Gene
ChrM
D
H Strand
Y=0.065X; p=2.7e-116
Methylation Level
L Strand
Y=0.18X; p=3.3e-63
Methylation Level
Starting Position of Each 50 bp Window on ChrM
L strand
Methylation Level
Regulatory
rRNA
tRNA
Gene
ChrM
E
PFC
Expression Level
ND1
ND2
CO1
CO2
ATP8
ATP6
CO3
ND3
ND4L
ND4
ND5
ND6
CYB

Supplemental Fig. 3. Methylation patterns and features across mtDNA in human brain.

(A) Heat maps of correlation (CSI) between mean methylation level of each functional element across 14 human PFC samples within the H strand or L strand. Functional elements are ordered based on their position on the reference genome (rCRS).

(B) Heat map of correlation (CSI) between mean methylation levels on each pair of functional elements for 14 human PFC samples including regulatory regions (red), tRNA (blue), rRNA (yellow) and protein coding gene (green) within H or L strand. Elements are hierarchically clustered.

(C) Mean methylation levels on each category of functional element for 14 human PFC on the H and L strand. Error bars represent the SEM among functional elements in PFC samples.

(D) Range of DNA methylation levels for 14 human PFC samples across mtDNA per 50 bp on the H strand and L strand. Vertical lines (grey) show the 25th percentile and 75th percentile of methylation level across PFC samples, and stars represent the mean of the methylation level across 14 human PFC samples.

(E) Mitochondrial transcript abundance levels in reads per kilobase per million mapped reads (rpkm). Error bars represent SEM among 9 PFC samples.

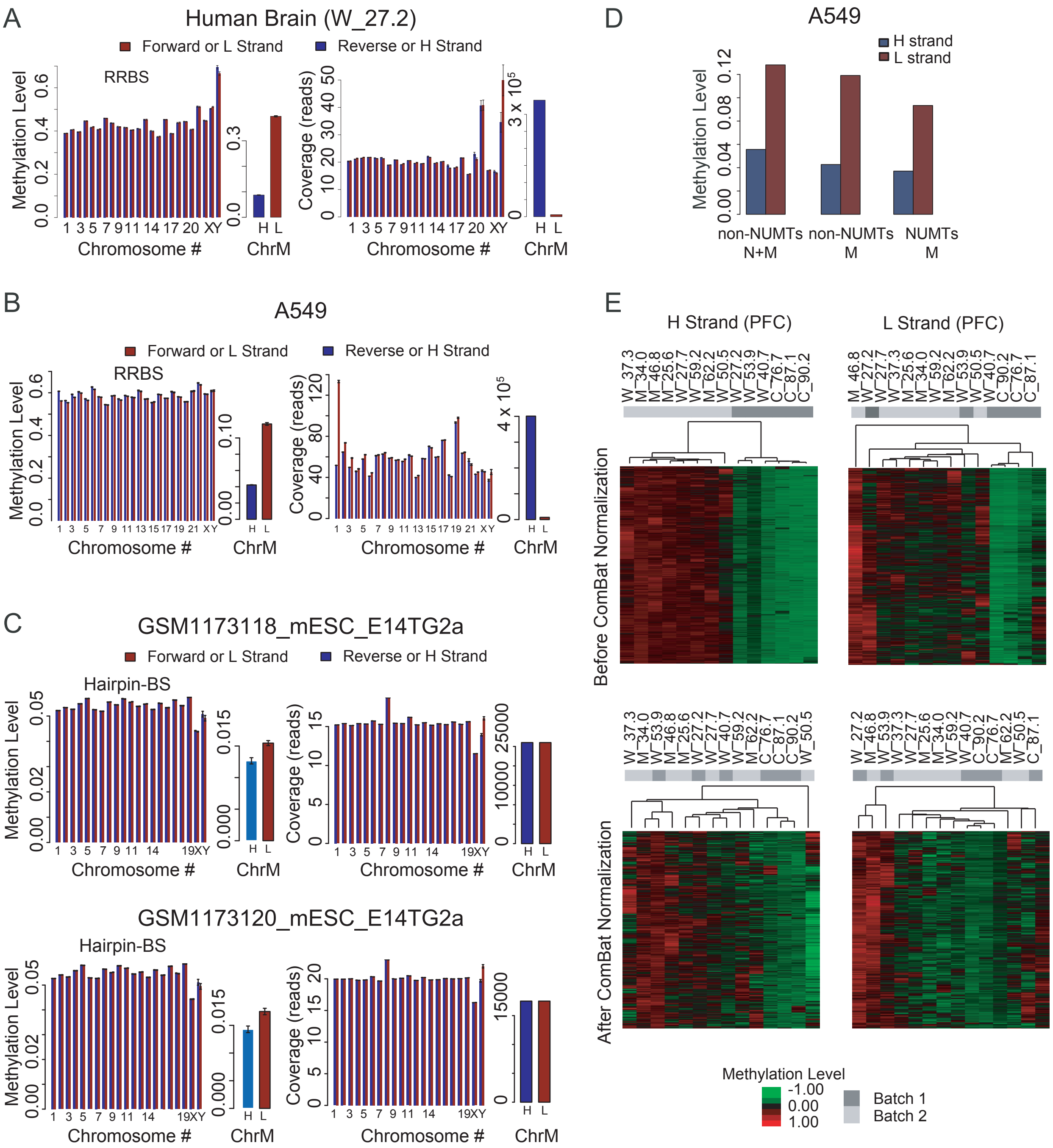

A
Human Brain (W_27.2)
Forward or L Strand
Reverse or H Strand
RRBS
Methylation Level
Coverage (reads)
Chromosome #
ChrM
B
A549
RRBS
C
GSM1173118_mESC_E14TG2a
Hairpin-BS
GSM1173120_mESC_E14TG2a
D
A549
H strand
L strand
non-NUMTs N+M
non-NUMTs M
NUMTs M
E
H Strand (PFC)
L Strand (PFC)
Before ComBat Normalization
After ComBat Normalization
Methylation Level
-1.00
0.00
1.00
Batch 1
Batch 2

Supplemental Fig. 4. Evaluating different WGBS methods in detection of mtDNA methylation.

(A) Methylation levels and sequence depth across all chromosomes and mtDNA in a representative PFC sample. Error bars represent the SEM among methylated CpG sites in autosomes or methylated C sites in ChrM.

(B) Methylation levels and sequence depth across all chromosomes and mtDNA in A549 cells. Error bars represent the SEM among methylated CpG sites in autochromosomes or methylated C sites in ChrM.

(C) Methylation levels and sequence depth across all chromosomes and mtDNA for two mouse ESC samples (d0 and d6 obtained from published datasets GSE48229). Error bars represent the SEM among methylated CpG sites for autosomes or methylated C sites for ChrM.

(D) Methylation level of non-NUMTs mapping with N+M (nucleus plus mitochondria) or only M (mitochondria), NUMTS mapping with M in A549 cells on the H and L strand separately. Error bars represent SEM among C sites covered by at least ten reads.

(E) Hierarchical clustering of PFC samples (columns) based on the methylation level of each C site on the H strand (up) and L strand (bottom) before and after ComBat normalization. Rows are Z-score normalized, and columns are clustered based on Euclidean distance.

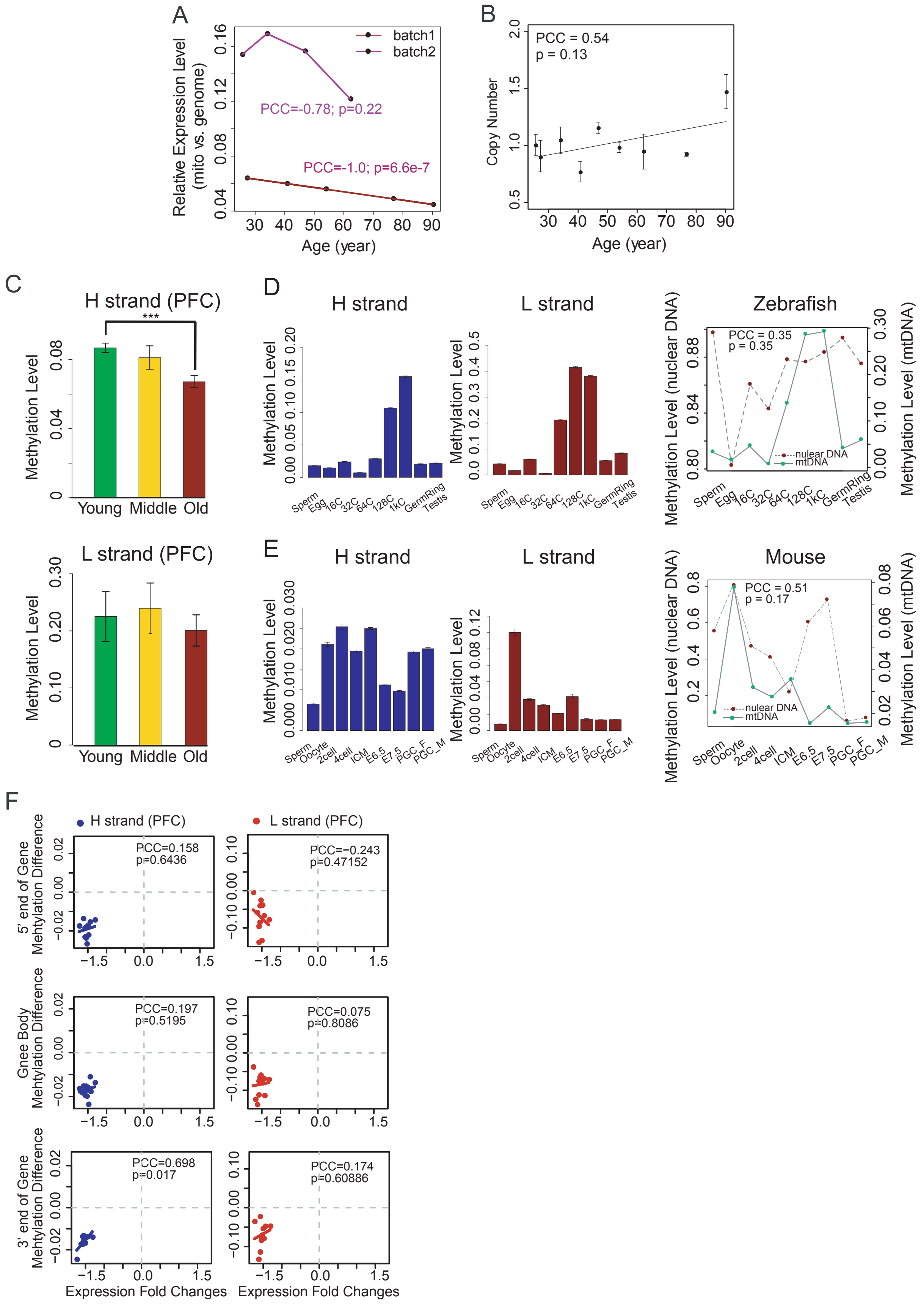
A
batch1
batch2
PCC=-0.78; p=0.22
PCC=-1.0; p=6.6e-7
Relative Expression Level (mito vs. genome)
Age (year)
B
PCC = 0.54
p = 0.13
Copy Number
Age (year)
C
H strand (PFC)
***
Methylation Level
Young Middle Old
L strand (PFC)
Methylation Level
Young Middle Old
D
H strand
L strand
Zebrafish
PCC = 0.35
p = 0.35
nulear DNA
mtDNA
Methylation Level
Methylation Level (nuclear DNA)
Methylation Level (mtDNA)
Sperm Egg 16C 32C 64C 128C 1kC GermRing Testis
E
H strand
L strand
Mouse
PCC = 0.51
p = 0.17
nulear DNA
mtDNA
Methylation Level
Methylation Level (nuclear DNA)
Methylation Level (mtDNA)
Sperm Oocyte 2cell 4cell ICM E6.5 E7.5 PGC_F PGC_M
F
H strand (PFC)
L strand (PFC)
5' end of Gene Mehtylation Difference
PCC=0.158
p=0.6436
PCC=−0.243
p=0.47152
Gnee Body Mehtylation Difference
PCC=0.197
p=0.5195
PCC=0.075
p=0.8086
3' end of Gene Mehtylation Difference
PCC=0.698
p=0.017
PCC=0.174
p=0.60886
Expression Fold Changes
Expression Fold Changes

Supplemental Fig. 5. mtDNA methylation decreases during aging and is negatively correlated with gene expression.

(A) Relative mean mitochondrial transcript abundance with age, based on RNA-seq data for 9 of the 14 PFC samples, and normalized by copy number shown in (B).

(B) mtDNA copy number over age range of samples. Error bars represent the SEM of three technical replicates.

(C) Mean methylation levels for samples grouped into young (20-40 years), middle age (40-60 years), and old (>60 years) on the H and L strand. *** indicates one sided *t*-test p value < 0.001. Error bars represent SEM within young (5 PFC samples), middle age (4 PFC samples) and old (5 PFC samples).

(D, E) Mean methylation on mtDNA H (left panel) and L (middle panel) strands, and nuclear DNA (right panel), respectively, during zebrafish (D) and mouse (E) early embryogenesis. Error bars represent SEM among all detected C sites.

(F) Scatter plot of expression fold changes (FC) against H and L strand methylation difference of 5' end (+/-200bp of TSS), gene body and 3' end (+/- 200bp of TTS) of genes comparing Old with Young.

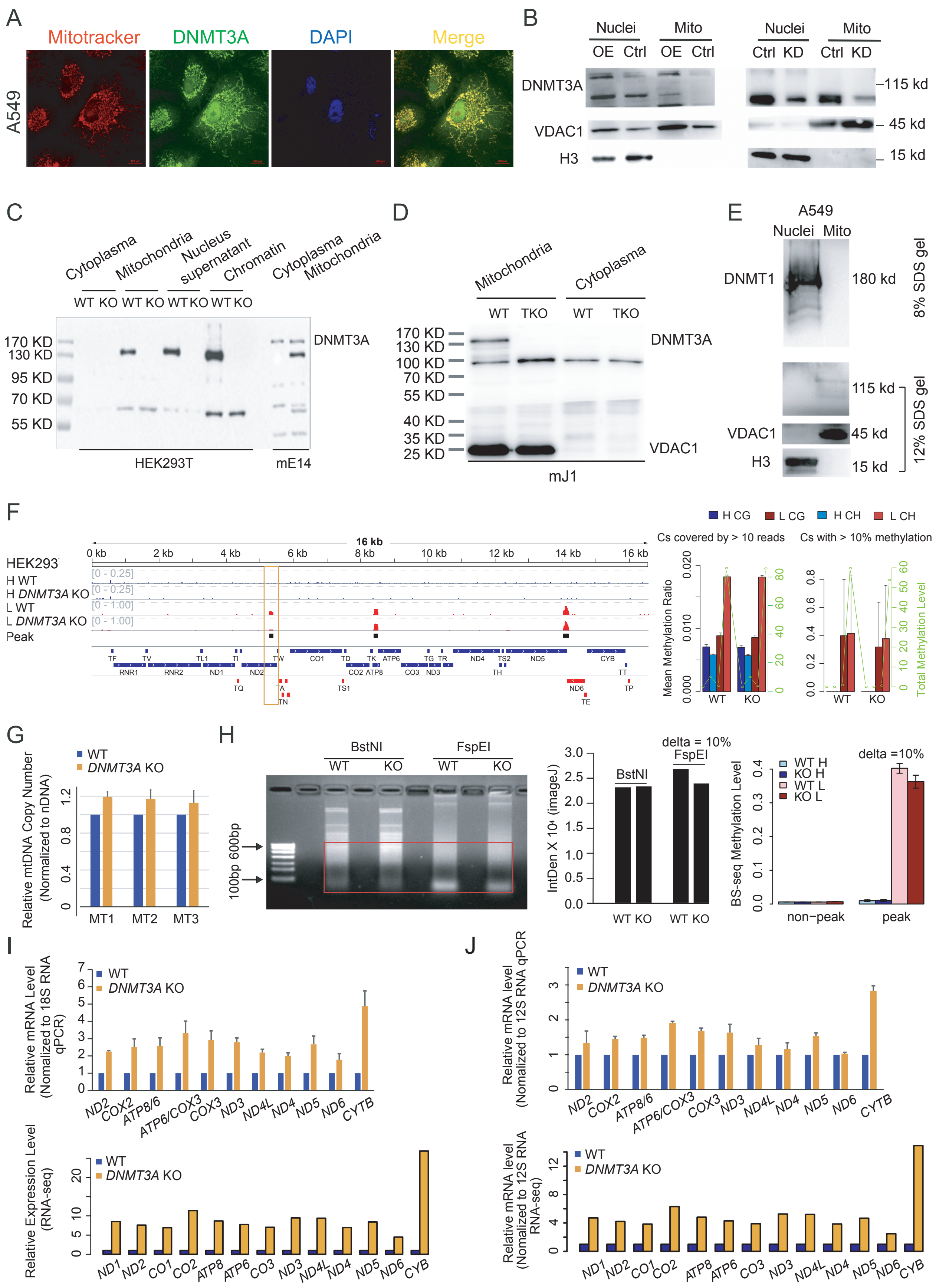
A
Mitotracker
DNMT3A
DAPI
Merge
A549
B
Nuclei
Mito
OE Ctrl OE Ctrl
Ctrl KD Ctrl KD
DNMT3A
VDAC1
H3
115 kd
45 kd
15 kd
C
Cytoplasma
Mitochondria
Nucleus supernatant
Chromatin
WT KO WT KO WT KO WT KO
170 KD
130 KD
95 KD
70 KD
55 KD
DNMT3A
HEK293T
mE14
D
Mitochondria
Cytoplasma
WT TKO WT TKO
25 KD
35 KD
40 KD
100 KD
VDAC1
mJ1
E
A549
DNMT1
180 kd
8% SDS gel
12% SDS gel
F
HEK293
H WT
H DNMT3A KO
L WT
L DNMT3A KO
Peak
H CG
L CG
H CH
L CH
Cs covered by > 10 reads
Cs with > 10% methylation
Mean Methylation Ratio
Total Methylation Level
G
Relative mtDNA Copy Number (Normalized to nDNA)
DNMT3A KO
MT1
MT2
MT3
H
BstNI
FspEI
100bp 600bp
delta = 10%
IntDen X 10⁴ (imageJ)
BS-seq Methylation Level
WT H
KO H
WT L
KO L
delta =10%
non−peak
peak
I
Relative mRNA Level (Nomalized to 18S RNA qPCR)
Relative Expression Level (RNA-seq)
J
Relative mRNA level (Nomalized to 12S RNA qPCR)
Relative mRNA level (Nomalized to 12S RNA RNA-seq)

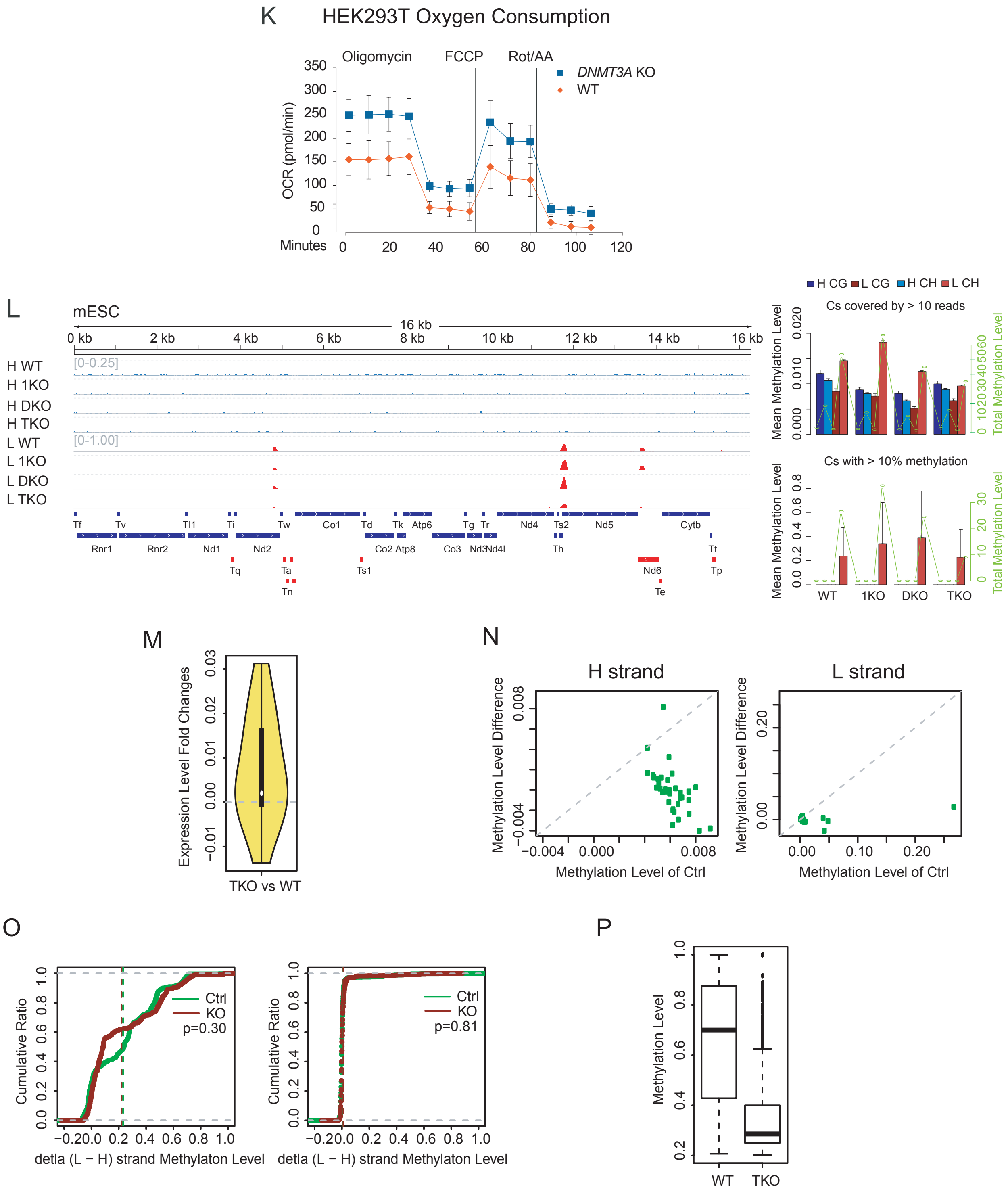
K
HEK293T Oxygen Consumption
Oligomycin
FCCP
Rot/AA
DNMT3A KO
WT
OCR (pmol/min)
Minutes
L
mESC
16 kb
H WT
H 1KO
H DKO
H TKO
L WT
L 1KO
L DKO
L TKO
[0-0.25]
[0-1.00]
Tf
Tv
Tl1
Ti
Tw
Co1
Td
Tk
Atp6
Tg
Tr
Nd4
Ts2
Nd5
Cytb
Rnr1
Rnr2
Nd1
Nd2
Co2
Atp8
Co3
Nd3
Nd4l
Th
Tt
Tq
Ta
Ts1
Nd6
Tp
Tn
Te
H CG
L CG
H CH
L CH
Cs covered by > 10 reads
Cs with > 10% methylation
Mean Methylation Level
Total Methylation Level
WT
1KO
DKO
TKO
M
Expression Level Fold Changes
TKO vs WT
N
H strand
L strand
Methylation Level Difference
Methylation Level of Ctrl
O
Cumulative Ratio
Ctrl
KO
p=0.30
p=0.81
detla (L − H) strand Methylaton Level
P
Methylation Level
WT
TKO

Supplemental Fig. 6. Effect of *DNMT3A* KO on mtDNA copy number and gene expression.

(A) Immunofluorescence staining of DNMT3A. Cells were exposed to MitoTracker Orange (red) to label mitochondria and anti-DNMT3A (green) and DAPI (blue) to label nuclei.

(B) Western blots of DNMT3A in *DNMT3A* OE (left panel) and KD (right panel) A549 cells and their corresponding controls. Cells were separated into nuclear and mitochondria fractions, and probed with anti-DNMT3A and VDAC1 as an internal mitochondria protein control and H3 as a nuclear protein control.

(C) Western blots of DNMT3A in both WT and *DNMT3A* knockout HEK293T cells, WT mouse E14. WT and *DNMT3A* KO HEK293T cell were separated into different components including cytoplasm, mitochondria, nucleus supernatant and chromatin, and mouse E14 cells were separated into cytoplasm and mitochondria, and probed with anti-DNMT3A.

(D) Western blots of DNMT3A in WT and TKO mouse J1 cells. J1 cells were separated into mitochondria and cytoplasm fractions, and probed with anti-DNMT3A and VDAC1 as an internal mitochondria protein control.

(E) Western blots of DNMT1 in WT A549. A549 cell were separated into nuclei and mitochondria, and probed with anti-DNMT1, and VDAC1 as an internal mitochondria protein control and H3 as nuclei control.

(F) Left panel, mtDNA methylation profiles of WT and *DNMT3A* KO HEK293T cell on the H and L strand. Blue tracks show H strand methylation levels (scale from 0 to 0.25) and red tracks show L strand methylation (scale from 0 to 1). Middle annotations show mtDNA elements: in blue are polypeptide genes, rRNA and tRNAs coded on H strand; in red are polypeptide genes and tRNAs coded on L strand. Right panel, summary statistics as annotated in Figure. 1.

(G) Relative copy number of mtDNA in WT and *DNMT3A* KO HEK293T cells. Error bars represent standard error among the three replicates.

(H) Gel electrophoresis of mtDNA (ug/reaction) digested by BstNI and FspEI in WT and *DNMT3A* KO HEK293T cells (left panel). Quantification (IntDen) of DNA smaller than 600 bp using ImageJ (middle penel). Mean mtDNA methylation of WT and *DNMT3A* KO HEK293T cells in non-peak and peak (F) regions on H or L strand. Error bars represent SEM among all detected C sites (right panel).

(I) Mitochondria gene expression level relative to 18S RNA by qPCR (top panel) and log_2 transformed RPKM by RNA-seq (bottom panel) in WT and *DNMT3A* KO HEK293T cells. Primers are listed in Table S8.

(J) Mitochondria gene expression level relative to 12S rRNA in WT and *DNMT3A* KO HEK293T cells by qPCR (top panel) and RNA-seq (bottom panel). Primers are listed in Table S8.

(K) Oxygen consumption of *DNMT3A* KO with WT HEK293T cells. Oxygen consumption represents basal respiration, followed by the respiration after ATP synthase inhibition by oligomycin, maximal respiration induced by FCCP, and total block of mitochondrial-respiration by rotenone/antimycin A (Rot/AA).

(L) Left panel, mtDNA methylation profiles of WT, *Dnmt1* KO (single KO, KO), *Dnmt3a*/*Dnmt3b* KO (double KO, DKO), and *Dnmt1*/*Dnmt3a*/*Dnmt3b* knockout (triple KO, TKO) mouse ESC on the H or L strand. Blue tracks show H strand methylation levels (scale from 0 to 0.25) and red tracks show L strand methylation (scale from 0 to 1). Middle annotations show mtDNA elements: in blue are polypeptide genes, rRNAs and tRNAs coded on H strand; in red are polypeptide genes and tRNAs coded on L strand. Right panel, summary statistics as annotated in Figure. 1.

(M) Violin plot of mitochondrial gene expression level relative to 12S rRNA changes comparing TKO with WT J1 mESCs.

(N) Scatter plot between mean methylation level of 63 functional elements of control samples (x-axis) and corresponding methylation level difference between *DNMT3A* KO and control samples (y-axis) on H and L strand.

(O) Cumulative curve of methylation level difference between L and H strand of C sites that contain methylation sum of Ctrl and KO > 0.05 (left panel) or > 0 (right panel) after sampling 1000 paired Cs under *DNMT3A* KO. The p value for the difference between *DNMT3A* KO and control was calculated with a one-sided *t*-test.

(P) Methylation levels of CpG sites with methylation ratio > 0.2 and coverage >5 in both WT and TKO J1 mESC on nuclear DNA.

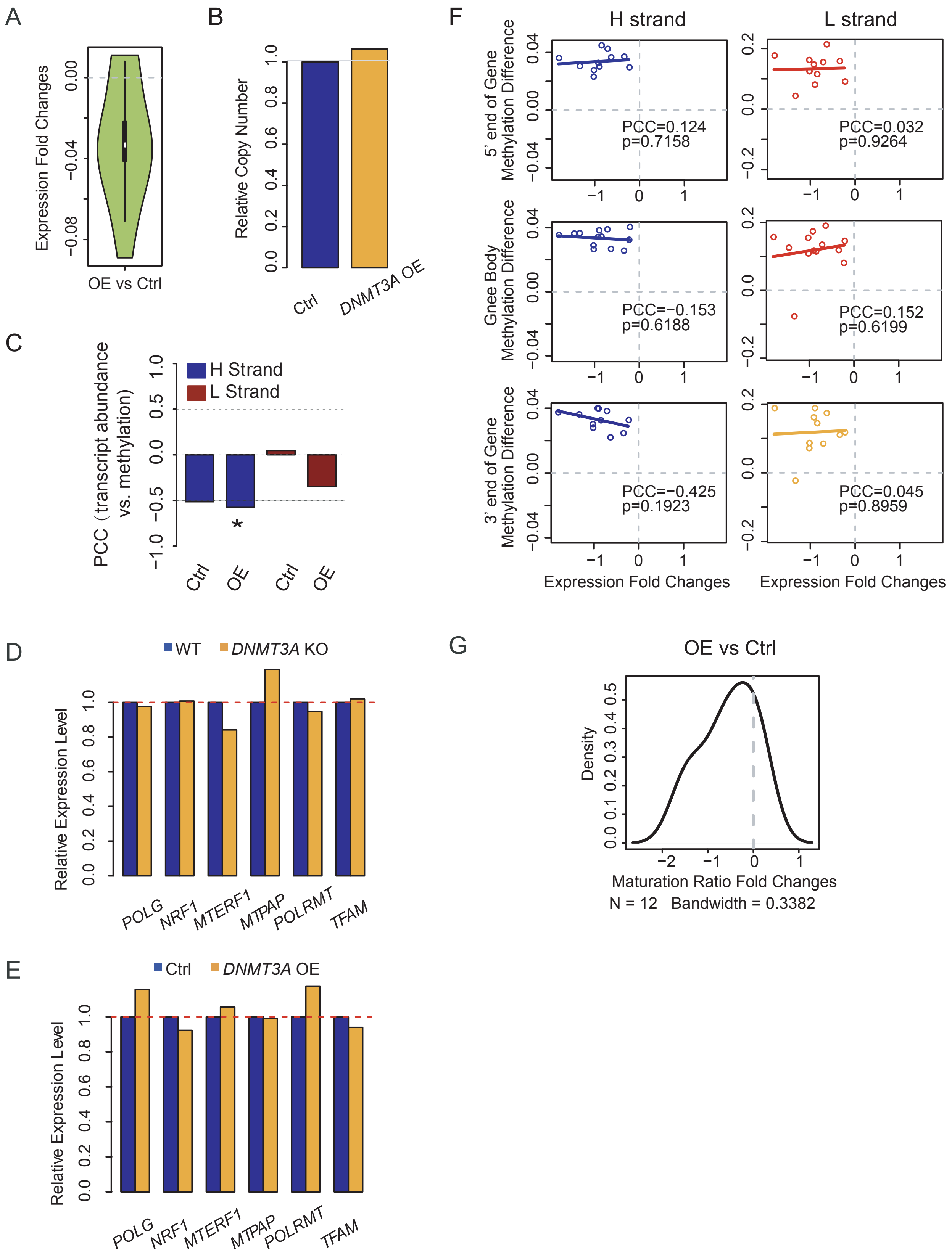

A
Expression Fold Changes
0.00
−0.04
−0.08
OE vs Ctrl
B
Relative Copy Number
0.0 0.2 0.4 0.6 0.8 1.0
Ctrl
DNMT3A OE
C
PCC（transcript abundance vs. methylation)
1.0 0.5 0.0 −0.5 −1.0
H Strand
L Strand
*
Ctrl OE Ctrl OE
D
WT
DNMT3A KO
Relative Expression Level
0.0 0.2 0.4 0.6 0.8 1.0
POLG NRF1 MTERF1 MTPAP POLRMT TFAM
E
Ctrl
DNMT3A OE
Relative Expression Level
0.0 0.2 0.4 0.6 0.8 1.0
POLG NRF1 MTERF1 MTPAP POLRMT TFAM
F
H strand
L strand
5' end of Gene Methylation Difference
PCC=0.124
p=0.7158
PCC=0.032
p=0.9264
Gnee Body Methylation Difference
PCC=−0.153
p=0.6188
PCC=0.152
p=0.6199
3' end of Gene Methylation Difference
PCC=−0.425
p=0.1923
PCC=0.045
p=0.8959
Expression Fold Changes
Expression Fold Changes
G
OE vs Ctrl
Density
Maturation Ratio Fold Changes
N = 12 Bandwidth = 0.3382

Supplemental Fig. 7. Effect of *DNMT3A* OE on mtDNA copy number and gene expression.

(A) Violin plot of expression fold changes comparing *DNMT3A* OE with control A549 cells.

(B) Relative mtDNA copy number in *DNMT3A* OE with control A549 cells. mtDNA copy number was estimated by reads mapped to mtDNA relative to nuclear DNA after normalizing their nucleotides coverage, respectively.

(C) Correlation between transcript abundance and mean gene body methylation level across genes within each sample on the H and L strand.

(D-E) Expression level of key nuclear encoded mitochondrial regulatory factors comparing *DNMT3A* KO (D) and *DNMT3A* OE (E) with their corresponding controls in HEK293T and A549 cells respectively.

(F) Scatter plot of expression fold changes (FC) against H and L strand methylation difference of 5' end (+/-200bp of TSS), gene body and 3' end (+/- 200bp of TTS) of gene comparing *DNMT3A* OE with control. * denotes negative PCC with $p < 0.01$.

(G) Density plot of maturation ratio fold changes comparing between *DNMT3A* OE with control.
